# Transcriptional profiling aligned with *in situ* expression image analysis reveals mosaically expressed molecular markers for GABA neuron sub‐groups in the ventral tegmental area

**DOI:** 10.1111/ejn.14534

**Published:** 2019-08-16

**Authors:** Eleanor J. Paul, Kyoko Tossell, Mark A. Ungless

**Affiliations:** ^1^ MRC London Institute of Medical Sciences (LMS) London UK; ^2^ Institute of Clinical Sciences (ICS) Faculty of Medicine Imperial College London London UK

**Keywords:** aversion, dopamine, limbic, midbrain, reward

## Abstract

γ‐Aminobutyric acid (GABA) neurons in the ventral tegmental area (VTA) provide local inhibitory control of dopamine neuron activity and send long‐range projections to several target regions including the nucleus accumbens. They play diverse roles in reward and aversion, suggesting that they be comprised of several functionally distinct sub‐groups, but our understanding of this diversity has been limited by a lack of molecular markers that might provide genetic entry points for cell type‐specific investigations. To address this, we conducted transcriptional profiling of GABA neurons and dopamine neurons using immunoprecipitation of tagged polyribosomes (RiboTag) and RNAseq. First, we directly compared these two transcriptomes in order to obtain a list of genes enriched in GABA neurons compared with dopamine neurons. Next, we created a novel bioinformatic approach, that used the PANTHER (Protein ANalysis THrough Evolutionary Relationships) gene ontology database and VTA gene expression data from the Allen Mouse Brain Atlas, from which we obtained 6 candidate genes: *Cbln4, Rxfp3, Rora, Gpr101, Trh* and *Nrp2*. As a final step, we verified the selective expression of these candidate genes in sub‐groups of GABA neurons in the VTA (and neighbouring substantia nigra pars compacta) using immunolabelling. Taken together, our study provides a valuable toolbox for the future investigation of GABA neuron sub‐groups in the VTA.

## Graphical abstract

GABA neurons in the ventral tegmental area (VTA) play diverse roles in reward and aversion, but our understanding of this diversity has been limited by a lack of sub‐group‐specific molecular markers. We conducted transcriptional profiling of GABA neurons, using immunoprecipitation of tagged polyribosomes (RiboTag) and RNAseq, combined with a novel bioinformatic approach, and immunolabelling, to identify several markers of GABA neuron subgroups in the VTA, including *Cbln4, Rxfp3, Rora and Gpr101*.

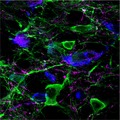

**Abbreviations**
AADC: Aromatic L‐amino acid decarboxylase
AMBA: Allen Mouse Brain Atlas
CLBN4: cerebellin 4 precursor
DAT: dopamine transporter
DRN: dorsal raphe nucleus
EN2: engrailed 2
GABA: γ‐aminobutyric acid
GABAARα1: α1 subunit of the GABAA receptor
GAD: glutamic acid decarboxylase
GATA3: GATA‐binding protein 3
GPR101: G protein‐coupled receptor 101
HA: hemagglutinin
IP: immunoprecipitation
ISH: in situ hybridisation
nNOS: neuronal nitric oxide synthase
NRP2: neuropilin 2
NTS: neurotensin
OCT: optimal cutting temperature
PAG: periaqueductal grey
PANTHER: Protein ANalysis THrough Evolutionary Relationships
PBP: parabrachial pigmented part of the VTA
PBS: phosphate‐buffered saline
PFA: paraformaldehyde
PN: paranigral part of the VTA
RNA: ribonucleic acid
RNAseq: RNA sequencing
RORA: RAR‐related orphan receptor A
RXFP3: relaxin/insulin‐like family peptide receptor 3
S100B: S100 calcium‐binding protein B
SNC: substantia nigra pars compacta
SST: somatostatin
TH: tyrosine hydroxylase
TRH: thyrotropin‐releasing hormone
VGAT: vesicular GABA transporter
VGLUT2: vesicular glutamate transporter 2
VTA: ventral tegmental area

## 1 INTRODUCTION

γ‐Aminobutyric acid (GABA) neurons make up around one‐third of neurons in the ventral tegmental area (VTA) (Nair‐Roberts et al., 2008; Olson & Nestler, 2007). These inhibitory neurons send long‐range axonal projections to several regions, including the nucleus accumbens (NAc) and ventral pallidum, and make local synaptic connections with dopamine neurons (Breton et al., 2018; Omelchenko & Sesack, 2009; Taylor et al., 2014). Optogenetic excitation of their local synaptic terminals can generate a conditioned place aversion and inhibits food consumption (Tan et al., 2012; van Zessen, Phillips, Budygin, & Stuber, 2012). It has been suggested that some VTA GABA neurons signal expected reward, and consequently, their excitation (in the absence of an actual reward) will generate a negative prediction error (i.e. an outcome worse than expected) and hence may be aversive (Cohen, Haesler, Vong, Lowell, & Uchida, 2012). Interestingly, it appears that VTA GABA neurons can limit wakefulness, at least in part by inhibiting dopamine neurons (Takata et al., 2018; Yu et al., 2019). In addition to the effects of GABA neuron stimulation within the VTA, optogenetic excitation of VTA‐>NAc projecting GABA neuron terminals can regulate associative learning, and excitation of VTA‐> dorsal raphe nucleus (DRN) and VTA‐> periaqueductal grey (PAG) projecting GABA neuron terminals elicits a cardiovascular depressor response (Brown et al., 2012; Kirouac, Li, & Mabrouk, 2004; Takata et al., 2018; Tan et al., 2012; van Zessen et al., 2012) and can interfere with morphine‐induced place preference (Li et al., 2019). Lastly, some GABA neurons in the VTA are targets of addictive drugs. For example, one key mechanism by which opiates have their rewarding effects in the VTA is through direct inhibition of a subset of GABA neurons, leading to disinhibition of dopamine neurons (Fields & Margolis, 2015).

Given these apparently diverse roles of GABA neurons in the VTA, it seems likely that this population is comprised of several functionally and anatomically heterogeneous sub‐groups. However, it has not been clear even whether amongst this population of GABA neurons there are dedicated local interneurons (as are seen in many other brain regions; Klausberger & Somogyi, 2008; Tepper, Tecuapetla, Koos, & Ibanez‐Sandoval, 2010; Kepecs & Fishell, 2014; Tremblay, Lee, & Rudy, 2016) or whether they are all projection neurons (that also make local synaptic connections). However, we have recently found that a sub‐group of GABA neurons in the VTA that express nNOS do not send axonal projections outside of the VTA suggesting that they are dedicated interneurons (Paul et al., 2018). Progress in further exploring diversity within this GABA neuron population is hindered by a lack of molecular markers identifying sub‐groups that could provide genetic entry points for cell type‐specific anatomical, physiological and behavioural studies. In particular, many of the classical molecular markers used to identify and selectively target sub‐groups of interneurons in other regions of the brain are either not expressed in the VTA or there is evidence suggesting that they are expressed by some dopamine neurons (e.g. somatostatin, cholecystokinin, vasoactive intestinal peptide, neuropeptide Y, parvalbumin and calretinin) (Dougalis et al., 2012; Gonzalez‐Hernandez & Rodriguez, 2000; Hokfelt et al., 1980; Isaacs & Jacobowitz, 1994; Klink, de Kerchove d'Exaerde, Zoli, & Changeux, 2001; Lein et al., 2007; Liang, Sinton, & German, 1996; Merrill, Friend, Newton, Hopkins, & Edwards, 2015; Olson & Nestler, 2007; Rogers, 1992; Seroogy et al., 1988, 1989). We, therefore, took an unbiased approach to identify novel, molecular candidates. We used the RiboTag methodology (Sanz et al., 2009) to obtain transcripts enriched in either GABA or dopamine neurons populations in the VTA. We then conducted RNAseq and identified a number of candidate markers by taking a novel bioinformatic approach, which used the PANTHER (Protein ANalysis THrough Evolutionary Relationships) gene ontology database followed by aligning RNAseq data with *in situ hybridisation* (ISH) expression data (from the Allen Mouse Brain Atlas (AMBA; Lein et al., 2007). We then tested a number of candidates using immunofluorescence, revealing several molecular markers for sub‐groups of GABA neurons within the VTA.

## 2 MATERIALS AND METHODS

### 2.1 Animal maintenance and breeding

VGATCre (vesicular GABA transporter; RRID:IMSR_JAX:016962) and RPL22^HA^ (RiboTag; RRID:IMSR_JAX:011029) mice were purchased from the Jackson laboratory, and DATCre (dopamine transporter; Turiault et al., 2007) were gifted from Prof. Francois Tronche. Mice heterozygous for VGATCre (VGATCre +/−) or DATCre (DATCre +/−) were crossed with mice homozygous for RPL22^HA^ (RiboTag +/+) producing VGATCre +/− RiboTag +/− offspring (VGATCre:RiboTag) or DATCre +/− RiboTag +/− offspring (DATCre:RiboTag). All breeding and experimental procedures were conducted in accordance with the Animals (Scientific Procedures) Act of 1986 (UK) and approved by Imperial College London's Animal Welfare and Ethical Review Body. All mice were maintained in social groups of 2–4, where possible, with appropriate environmental enrichment (e.g. bedding and tunnels). They were kept in rooms at a constant temperature and maintained on a 12‐hr light/dark cycle. They were fed on standard rodent chow and water *ad libitum*.

### 2.2 Tissue fixation and preparation

VGATCre:RiboTag or DATCre:RiboTag mice were anaesthetised under isoflurane (4%) and given a lethal intraperitoneal (IP) injection of pentobarbital (100 mg/ml; Euthatal). They were transcardially perfused with 50 ml of ice‐cold phosphate‐buffered saline (PBS) followed by 50–100 ml of 4% paraformaldehyde (PFA; Sigma‐Aldrich) in PBS. When fixed, the brains were removed and placed in 10 ml of 4% PFA for 1 hr post‐fixation at room temperature. After three washes in PBS, brains were placed in 30% sucrose (Sigma‐Aldrich) dissolved in PBS for cryoprotection and kept at 4°C for 24–48 hr. Subsequently, brains were embedded in optimal cutting temperature (OCT) medium and snap‐frozen in isopentane (2‐methylbutane) at −55°C. All tissue was then stored at −80°C until sectioning.

### 2.3 Immunocytochemistry

All immunolabelling was conducted on tissue from male mice aged 8–12 weeks old. Brains were sectioned using a Leica CM1800 cryostat (Leica Microsystems, Germany). Coronal sections (70 μm for validating hemagglutinin (HA) expression and 30 μm for candidate staining) were taken from the midbrain of VGATCre:RiboTag or DATCre:RiboTag mice. Free‐floating sections were washed in PBS for 10 min at room temperature. Following this, they were blocked in 6% normal donkey serum (NDS) in 0.2% Triton‐x in PBS (PBSTx) for 60 min at room temperature. Primary antibodies (Table 1) were diluted in 2% NDS in PBSTx, and sections were incubated in the primary antibody solutions overnight at 4°C. Sections were washed (3 × 10 min) in PBS at room temperature. Secondary antibodies (Table 2) were diluted in 2% donkey serum 0.2% PBSTx. Sections were incubated in secondary antibody solution for a minimum of 1.5 hr at room temperature. They were then washed (3 × 10 min) in PBS. Stained sections were mounted onto glass microscope slides and when dry were cover‐slipped using VectaShield mounting medium (Vector Laboratories). The SNC and VTA sub‐regions were determined using tyrosine hydroxylase (TH) expression.

**Table 1:** Primary antibodies

Antibody	Host species	Supplier (product no.)	Concentration
Anti‐TH	Chicken	Abcam (ab76442; AB_1524535)	1:1,000
Anti‐HA	Mouse	Sigma (#H3663) AB_262051	1:1,000
Anti‐HA	Rabbit	Abcam (#ab9110) AB_307019	1:500
Anti‐GABAARα1	Rabbit	Alomone (#AGA‐001) AB_2039862	1:500
Anti‐GPR101	Rabbit	Sigma (#SAB4503289) AB_10761516	1:500
Anti‐NRP2	Rabbit	Cell Signalling (#D39A5) AB_2155250	1:200
Anti‐CBLN4	Rabbit	Invitrogen (#PA5‐36472) AB_2553539	1:500
Anti‐RORA	Rabbit	Sigma (#Av45608) AB_1856399	1:100
Anti‐TRH	Rabbit	Abcam (#ab185658)	1:100
Anti‐RXFP3	Goat	Santa Cruz (#sc47004) AB_2184857	1:100

**Table 2:** Secondary antibodies

Antibody	Conjugation	Host species	Supplier (product no.)	Concentration
Anti‐Chicken	Alexa‐488	Goat	Thermo Fisher Scientific (A11039; AB_2534096)	1:1,000
Anti‐Chicken	Cy3	Donkey	Jackson ImmunoResearch Labs (703‐165‐155; AB_2340363)	1:1,000
Anti‐Mouse	Cy3	Donkey	Jackson ImmunoResearch Labs (715‐165‐150: AB_2340813)	1:1,000
Anti‐Mouse	Cy5	Donkey	Jackson ImmunoResearch Labs (715‐175‐151; AB_2340820)	1:1,000
Anti‐Rabbit	Alexa‐633	Goat	Thermo Fisher Scientific (A21070; AB_2535731)	1:1,000
Anti‐Rabbit	Cy3	Donkey	Jackson ImmunoResearch Labs (711‐165‐152; AB_2307443)	1:1,000
Anti‐Goat	Alexa‐488	Donkey	Thermo Fisher Scientific (A11055; AB_2534102)	1:1,000
Anti‐Guinea Pig	Alexa‐488	Goat	Thermo Fisher Scientific (A11073: AB_2534117)	1:1,000

### 2.4 Microscopy

Confocal images were acquired using a Leica SP5 confocal microscope with the pinhole set at 1 Airy unit. All images were processed with Fiji software. Images were acquired with z‐stacks of 1 μm. To determine co‐localisation, channels were viewed both individually and in composite. Co‐localisation was determined if the cell body was visible in multiple channels through its entire thickness (multiple z planes). Representative examples of stacked images are shown.

### 2.5 Quantification of co‐expression

For the quantification of HA/ GABAARα1 co‐localisation or candidate‐expressing neurons, triple immunolabelling for GABAARα1/RORA/RXFP3/CBLN4/GPR101, HA and TH was conducted in male VGATCre:RiboTag mice (*n* = 3). To obtain estimates of the degree of co‐localisation between GABAARα1 each candidate with HA and TH, confocal images were processed using Fiji (ImageJ). Total numbers of GABAARα1/RORA/RXFP3/CBLN4/GPR101+ cells, HA+ cells and TH+ cells in each image were counted along with co‐expressing cells using the ImageJ cell counter plugin. These numbers were used to calculate the percentages indicating the degree of co‐expression and the proportions of candidate‐expressing sub‐populations.

### 2.6 Preparation of fresh tissue for immunoprecipitation

Ten‐ to 14‐week‐old mice were anaesthetised with isoflurane then decapitated. Their brains were removed and rapidly washed in ice‐cold PBS. The brains were placed on filter paper, and excess tissue was trimmed away with a razor blade leaving a block of tissue containing the ventral midbrain. The midbrain was then snap‐frozen in isopentane at −55°C. Tissue was stored at −80°C until use.

### 2.7 Immunoprecipitation of tagged polyribosomes

Hundred microliter of protein G magnetic beads were pipetted into a 1.2‐ml Eppendorf tube and placed on a magnet stand. The storage fluid was removed, and the beads were washed in 1 ml of citrate‐phosphate buffer, pH 5.0 at 4°C for 5 min. After washing, the beads were incubated with 10 μl of anti‐HA antibody (Sigma‐Aldrich #H6908; Table 1), and the solution was mixed by pipetting and placed on a rotator at 4°C for 45 min. After coupling, the beads were washed once in fresh citrate‐phosphate buffer, then twice in immunoprecipitation buffer (50 mM Tris, pH 7.5, 100 mM KCL, 12 mM MgCl_2_, 1% IGEPAL; Sanz et al., 2009).

Midbrain tissue was cryosectioned at 50 μm, and sections were collected with chilled forceps and placed in chilled 1.2‐ml Eppendorf tubes before being moved onto dry ice until homogenisation. 500 ml of polysome buffer (50 mM Tris, pH 7.5, 100 mM KCL, 12 mM MgCl_2_, 1% IGEPAL, 1 mM DTT, 200 U/ml Promega RNAsin, 1 mg/ml heparin, 100 mg/ml cycloheximide (Sigma‐Aldrich), 1× protease inhibitor cocktail (Sigma‐Aldrich) was added to each tube of sections. Sections were homogenised on ice using a motorised pestle mixer. Following homogenisation, an additional 500 ml of polysome buffer was added. Samples were centrifuged at 10,000 *g* for 10 min at 4°C to create a post‐mitochondrial supernatant (Sanz et al., 2009).

Following the final wash of the antibody‐coupled beads, the tubes were placed on a magnet, and the immunoprecipitation buffer was removed. 800 ml of supernatants were then added directly to the antibody‐coupled Protein G magnetic beads and rotated overnight at 4°C. 50 μl homogenate was placed in a fresh Eppendorf tube and placed on dry ice for later analysis. The following day, the samples were placed on the magnet and the supernatant was removed and collected. The beads were then washed three times in 1 ml of high salt buffer (50 mM Tris, pH 7.5, 300 mM KCl, 12 mM MgCl2, 1% IGEPAL, 1 mM DTT 100 μg/ml cycloheximide). Each time, the solutions were mixed by gentle pipetting and placed on a rotator at 4°C for 5 min. After the final wash, polysomes were eluted from the beads by adding 350 μl of lysis buffer (RLT buffer, Qiagen RNAeasy mini kit and 10 μl of β‐mercaptoethanol for every ml of RLT) and vortex mixing for 30 s. The eluted polysomes were removed from the beads and placed in a fresh 1.2‐ml Eppendorf tube on ice (Sanz et al., 2009).

### 2.8 RNA extraction, library preparation and sequencing

Three hundred fifty microlitre of lysis buffer was added to 50 μl of the input and unbound samples. The solutions were vortexed for 30 s then centrifuged for 3 min at 10,000 *g*. Three hundred fifty microlitre of 70% ethanol was added to the input, the unbound and the IP samples. The solutions were mixed by gentle pipetting, and RNA was extracted using an RNAmini extraction kit (QIAGEN). Following extraction, RNA was eluted from the columns in 30 μl of RNase and nuclease‐free H_2_O. RNA was then reverse transcribed for qPCR analysis, taken for total RNA analysis on the bioanalyzer or placed in dry ice and stored at −80°C for later use. The quality of RNA samples was assessed using an RNA integrity number (RIN), obtained through capillary gel electrophoresis with an Agilent Bioanalyzer and generated via an electropherogram using Agilent 2100 software. RNA samples with a RIN >7 were used to prepare RNA libraries. 150–200 ng of total RNA was used to prepare RNA libraries. Samples were first enriched for poly(A)‐containing transcripts using NEBNext Poly(A) mRNA Magnetic Isolation Module. Libraries were synthesised using NEBNext^®^ Ultra™ Directional RNA Library Prep Kit for Illumina^®^ and NEBNext^®^ Multiplex Oligos for Illumina^®^ (Index Primers Set 1). Libraries were synthesised based on manufacturer's instructions with the only difference being the volume of the total RNA sample and of the beads at the beginning of the process. Instead of 20 μl of NEBNext Oligo d(T)_25_ beads and 50 μl of diluted RNA sample, 40 μl of beads and 100 μl of diluted RNA sample were used in the mRNA isolation step. Prior to sequencing, the quality of the fragmented cDNA library was assessed on the Agilent bioanalyzer using a high sensitivity DNA chip. Libraries were diluted to equal molarity and sequenced (in triplicate) using an Illumina HiSeq 2500 system.

### 2.9 RNAseq analysis

Raw RNAseq reads were aligned against Ensembl Mouse genome reference sequence assembly (mm9) and transcript annotations using Tophat splice junction mapper version 2.0.11 (Kim et al., 2013). All parameters were set to default except inner distance between mate pairs (*r* = 100) and library type (fr‐firststrand). Gene‐based read counts were obtained using HTSeq count module (version 0.5.4p3) (Anders, Pyl, & Huber, 2015). This was run using parameters m = union, stranded = reverse. Differential expression analysis was performed using DESeq2 Bioconductor package (version 1.4.5) (Love, Huber, & Anders, 2014). The DESeq2 package uses a negative binomial model to model read counts and then performs statistical tests for differentially expression of genes. Raw *p* values were then adjusted for multiple testing with the Benjamini–Hochberg procedure. Genes with adjusted *p* value (*P‐Adj*) of 0.05 or less were considered differentially expressed. Quantitative *in situ* hybridisation (ISH) data for the VTA from the Allen Mouse Brain Atlas (AMBA) were obtained from: http://download.alleninstitute.org/informatics-archive/october-2014/mouse_expression/ (using custom‐written scripts: https://gitlab.com/mlinitaur/vta-expression-data-extractor).

## 3 RESULTS

**Figure 1:**
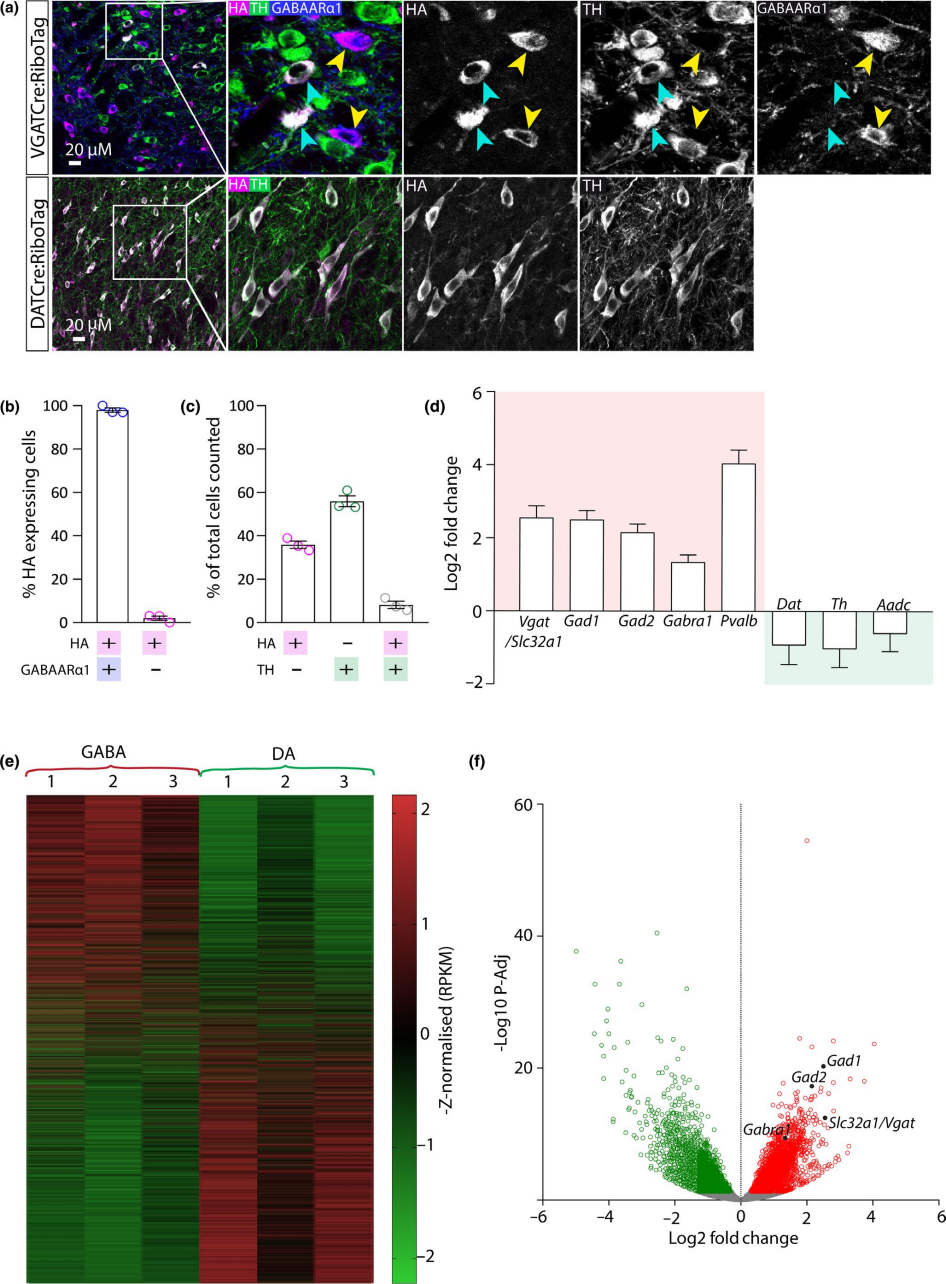
Isolation and sequencing of RNA from samples enriched for either GABA or dopamine neurons. (a) Representative images showing triple immunolabelling for HA, GABAARα1 and TH in the VTA from a VGATCre:RiboTag mouse (top panels) and double immunofluorescence for HA and TH in the VTA from a DATCre:RiboTag mouse (bottom panels). In each case HA immunoreactivity is selectively localised to the target neuronal population as illustrated by co‐localisation with GABAARα1 (in HA+/TH‐ cells (yellow arrows) but not in TH+ cells (blue arrows)) in the top panels and TH in the bottom panels. (b) Graph showing mean (+SEM) percentage of cells (*n* = 481) that were either HA+, TH+, or HA+ and TH+. (c) Graph showing mean (+SEM) percentage of HA+ (TH‐) cells (*n* = 278) that expressed GABAARα1. (d) Graph showing mean (+SEM) GABA neuron‐specific transcripts (red box; *slc32a1, Gad1, Gad2, Gabra1, Pvalb*) significantly enriched in samples enriched for GABA neurons compared to dopamine neurons (*P‐Adj* < 0.05). In contrast, dopamine neuron‐related transcripts *(*green box; *Dat, Th, Aadc)* were not enriched (*P‐Adj* > 0.05). (e) Heatmap depicting the 15,015 mRNA transcripts detected in GABA and dopamine neurons (each row represents a different transcript). Expression levels (reads per kilobase of transcript per million mapped reads; RPKM) were scaled and sorted by z‐score. The red‐green scale illustrates level of enrichment (red = enriched; green = de‐enriched). Each column represents a different RNA sample (biological repeat) for GABA (VGATCre:RiboTag) and dopamine (DA; DATCre:RiboTag). (f) Volcano plot illustrating genes that were differentially expressed (*p‐Adj* < 0.05) in GABA and dopamine neurons (each dot represents an individual gene). 7,530 genes were differentially expressed (green and red dots), 3,364 of which were significantly enriched in GABA neurons (red dots), including GABA neuron marker genes (*Gad1, Gad2, Gabra1 and Slc32a1*; labelled in black). [Colour figure can be viewed at http://www.wileyonlinelibrary.com]

To obtain tissue samples enriched for transcripts from GABA neurons, we used the RiboTag approach (Sanz et al., 2009). RiboTag mice have a hemagglutinin tagged exon 4 in the Rlp22 gene, which encodes for a core ribosomal subunit. The tagged exon lies downstream of the wild‐type (WT) exon 4, which is flanked by LoxP sites. Under normal conditions, the WT exon is expressed. However, in the presence of Cre‐recombinase, recombination occurs and the WT exon is excised resulting in expression of the HA‐tagged exon. HA‐tagged ribosomal protein is incorporated into polyribosomes, and these polyribosomes and any associated mRNA can then be immunoprecipitated out of a mixed cell homogenate using antibodies for the HA‐tag (Sanz et al., 2009). We first used immunofluorescence to confirm the selective expression of Rpl22‐HA in GABA neurons and dopamine neurons in the VTA of RiboTag mice crossed with either VGATCre or DATCre mice, respectively. In the VGATCre:RiboTag mice, we observed extensive co‐localisation between HA and GABAARα1 (the alpha 1 subunit of the GABAA receptor; a commonly used marker of GABA neurons in the VTA; Tan et al., 2012) and limited co‐localisation with TH (the rate‐limiting enzyme in dopamine synthesis, and the most commonly used marker of dopamine neurons in the VTA) (Figure 1a). In the DATCre:RiboTag mice, there was complete co‐localisation between HA and TH (Figure 1a). A quantification of neurons expressing HA, or TH, or HA and TH, in the VTA of VGATCre:RiboTag mice revealed proportions of these neurochemical sub‐groups (Figure 1b) similar to previous reports (e.g. Nair‐Roberts et al., 2008). Importantly for our subsequent immunolabelling validation, we observed near‐complete overlap between expression of GABAARα1 and HA (in TH‐ cells) indicating that they are GABAergic (Figure 1c; see Tan et al., 2012). Having confirmed the cell type‐specific expression of the HA‐tagged ribosomes in the VTA, we isolated blocks of tissue containing the ventral midbrain from VGATCre:RiboTag (GABA‐IP; *n* = 3) and DATCre:RiboTag (dopamine‐IP; *n* = 3) mice and conducted immunoprecipitations (IP) followed by RNA sequencing (RNAseq). To first validate our samples, we examined the fold enrichment levels of several GABA neuron‐specific and dopamine neuron‐specific genes, in our GABA‐IP samples relative to levels in dopamine‐IP samples. All GABA neuron‐specific transcripts examined were significantly enriched, whereas dopamine neuron‐specific transcripts were not (Figure 1b). Next, we conducted a full comparison of the libraries obtained from GABA‐IP and dopamine‐IP samples. A total of 15,014 genes were differentially expressed in the GABA‐IP samples compared with the dopamine‐IP samples. Importantly, the RNAseq results obtained within each group were similar across biological replicates, with slightly more genes appearing enriched in dopamine neurons than in GABA neurons (Figure 1c). A total of 3,364 genes were significantly enriched in the GABA‐IP samples compared with the dopamine‐IP samples (*P‐Adj* < 0.05), whereas 3,598 genes were significantly de‐enriched (Figure 1d), and 8,052 genes did not exhibit statistically significant differential expression.

**Figure 2:**
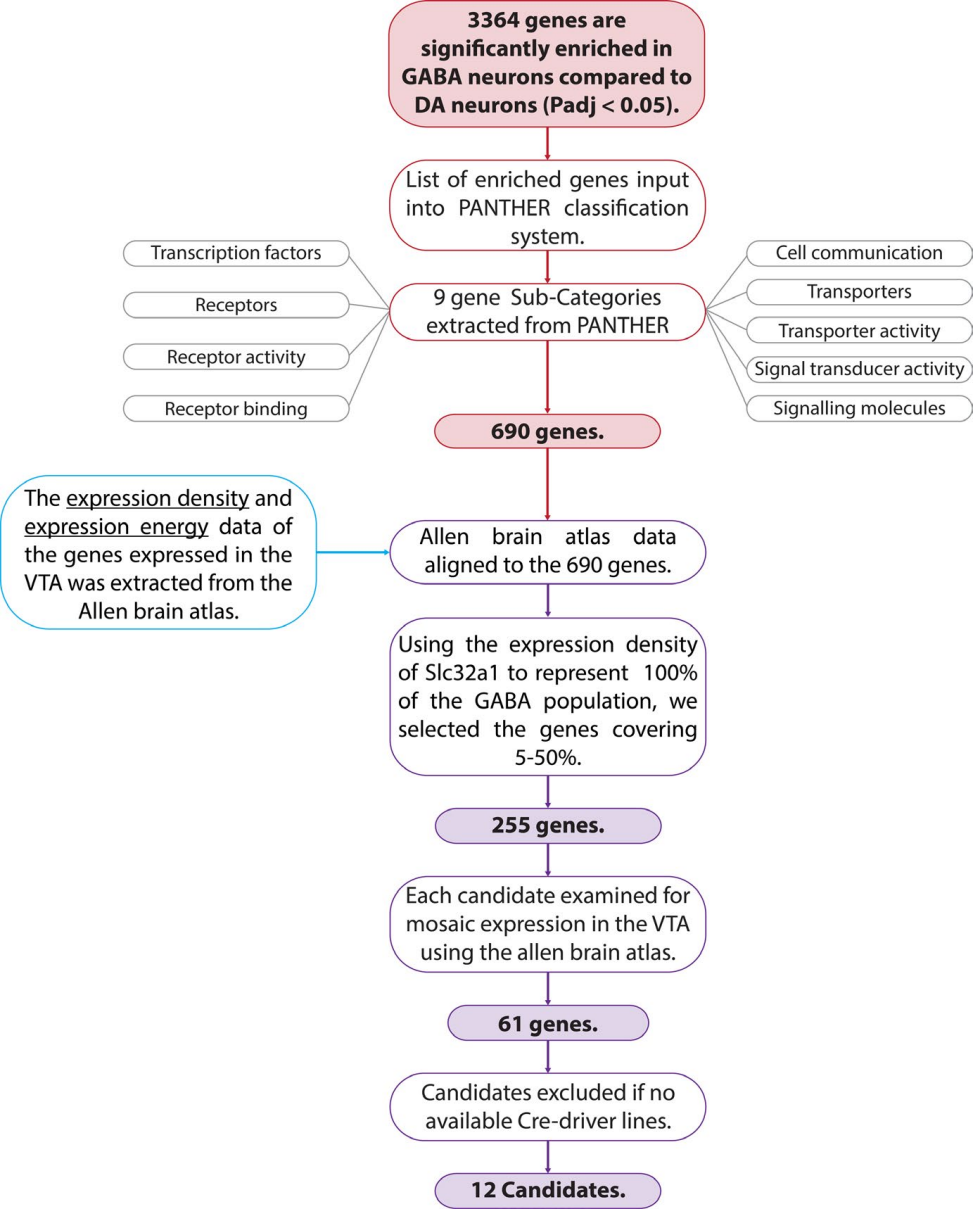
Flowchart depicting the pipeline designed to prioritise candidate genes for further investigation. [Colour figure can be viewed at http://www.wileyonlinelibrary.com]

Having identified many genes that were enriched in the GABA‐IP samples, we constructed a pipeline for the prioritisation of candidate genes for further analysis as markers of GABA neuron sub‐groups in the VTA. In order to avoid any potential biases when selecting candidates from the list of 3,364 genes significantly enriched in GABA neurons, we took a series of systematic steps (Figure 2). First, the list of enriched genes was input into the PANTHER (Protein ANalysis THrough Evolutionary Relationships) gene ontology database. Here, genes and their proteins are classified according to family and subfamily, molecular function, biological process or pathway (Mi, Muruganujan, Casagrande, & Thomas, 2013; Mi et al., 2017). Nine gene sub‐categories were extracted from PANTHER based on previous publications investigating molecular diversity within neuronal populations (Bikoff et al., 2016; Okaty et al., 2015; Paul et al., 2017), including transcription factors, cell communication, receptors, receptor activity and receptor binding, transporters, transporter activity, signal transducer activity and signalling molecules. In total, 690 genes were extracted from PANTHER (see Table S1).

**Figure 3:**
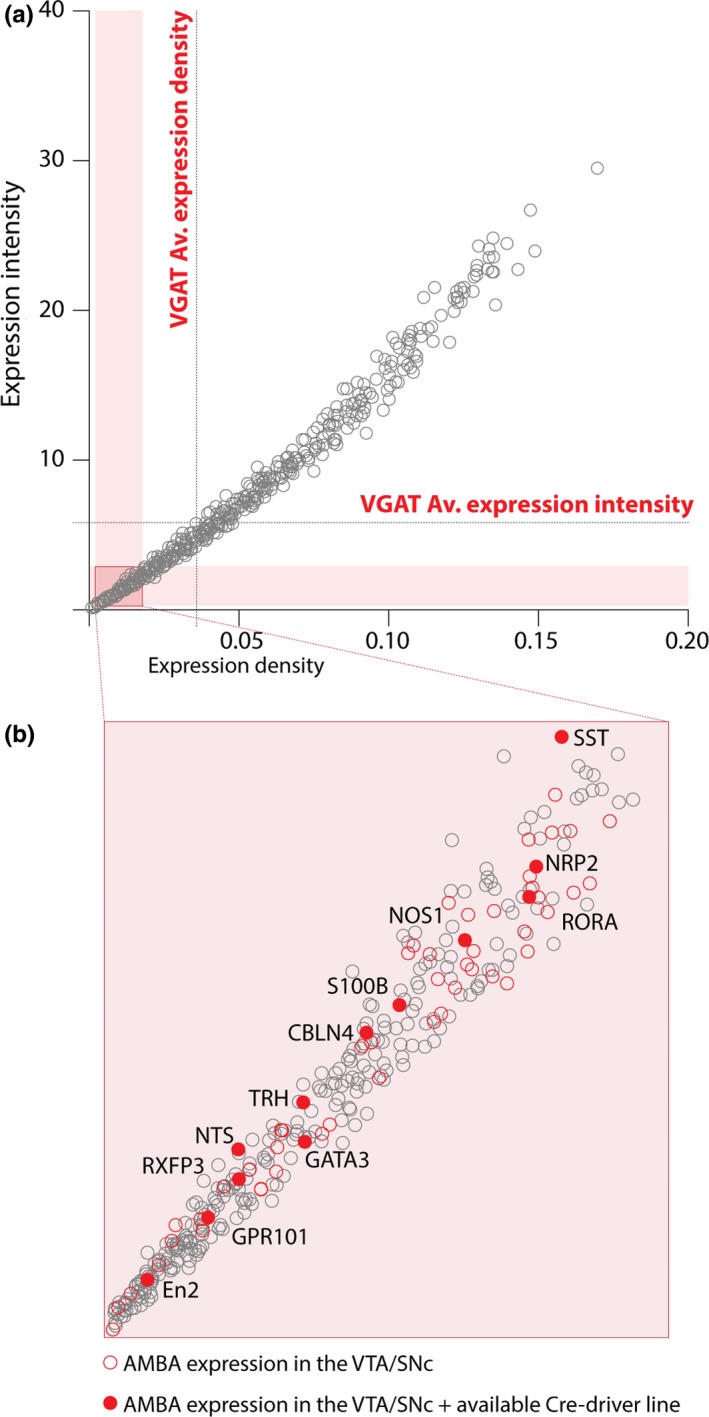
Allen Mouse Brain Atlas (AMBA) expression values for genes enriched in GABA neurons. a, Scatter plot showing AMBA expression density and expression intensity values for genes enriched in GABA neurons (*n* = 1,102; each grey circle represents a gene). Expression density = number expressing pixels/total number of pixels. Expression intensity = sum of expressing pixel intensity/sum of expressing pixels. Dotted lines illustrate the average values for VGAT (i.e. 100% of GABA neuron population). Pink boxes highlight 5%–50% of GABA neuron population. b, Scatter plot showing the highlighted pink box in A (i.e. 5%–50% of the GABA neuron population; *n* = 255 genes). Empty red circles represent genes that exhibit mosaic expression in ISH images in the AMBA. Filled red circles illustrate genes with both mosaic expression in the AMBA and available cre‐driver mouse lines. [Colour figure can be viewed at http://www.wileyonlinelibrary.com]

Next, we took a novel bioinformatic approach to identify genes that were both enriched in GABA neurons and expressed mosaically within the VTA. To do this, we aligned our RNAseq data with quantitative *in situ* hybridisation (ISH) data for the VTA from the Allen Mouse Brain Atlas (Figures 2 and 3a). Based on ISH images for each gene, expression density values correspond to the number of cells expressing each gene, and the gene expression level corresponds to the degree to which a gene is expressed by a given cells (Lein et al., 2007). In order to identify candidates that were expressed in subsets of GABA neurons, we used the expression density of *Vgat* (a gene expressed in all GABA neurons) to signify 100% of the population of GABA neurons (i.e. all genes with an expression density value higher than that of *Vgat* are likely to be expressed in all GABA neurons, or multiple cell types within the midbrain), and then, we extracted those genes expressed in between 5% and 50% of VTA GABA neurons (Figure 3b). This approach identified 255 genes that were enriched in our GABA‐IP samples and were expressed in the VTA, but at a density of 5%–50% of the whole GABA neurons population (see Table S1).

Next, we examined ISH images on the Allen Mouse Brain Atlas for each of the 255 candidate genes. We discarded genes without expression in the VTA or with widespread expression (e.g. in the SNpr and IPN). This left 61 genes. Because our ultimate aim was to identify genetic markers for the identification and the selective targeting and manipulation of sub‐populations of GABA neurons, we further refined this candidate list by discarding genes for which no cre‐driver mouse lines are available. This resulted in a list of 12 candidate genes that were enriched in our GABA‐IP samples, mosaically expressed in the VTA, and for which cre‐driver mouse lines exist (see Table S1). It is already well established that GATA‐binding protein 3 (GATA3) is expressed in all GABA neurons of the midbrain (Lahti, Achim, & Partanen, 2013; Lahti et al., 2016; Lakshmanan et al., 1999; Nardelli, Thiesson, Fujiwara, Tsai, & Orkin, 1999). In addition, there is evidence of expression of neurotensin (NTS) and engrailed 2 (EN2) in midbrain dopamine neurons (Hökfelt, Everitt, Theodorsson‐Norheim, & Goldstein, 1984; Seroogy et al., 1988; Simon, Saueressig, Wurst, Goulding, & O'Leary, 2001; Simon, Thuret, & Alberi, 2004; Studler et al., 1988) and S100b is a commonly used molecular marker of glial cells (Raponi et al., 2007) which might indicate some glial cell contamination in the IP sample or may simply be a false positive. For these reasons, these genes were not investigated further as potential candidates. Lastly, previous immunolabelling experiments had failed to reveal somatostatin (SST)‐positive cells within the VTA (possibly due to the antibodies not working well in the region). Our final list of seven candidate genes included neuronal nitric oxide synthase (NOS1), which provides a useful validation of our approach, because we have recently shown that it is expressed in a sub‐population of GABA neurons in the VTA (Paul et al., 2018). We next sought to validate the remaining six candidates (CBLN4, GPR101, RXFP3, RORA, NRP2 and TRH; Table 3) using immunolabelling.

**Table 3:** Candidate genes

Symbol	Gene name	Function	Log2 Fold Change	P‐Adj	Cre‐driver mouse
*Gpr101*	G protein‐coupled receptor 101	Orphan G protein‐coupled receptor	1.24	4.25E‐06	Reinius et al.*,* (2015)
*Cbln4*	Cerebellin 4 precursor	Small secreted protein	1.28	1.42E‐05	MMRRC
*Rxfp3*	Relaxin/insulin‐like family peptide receptor 3	G protein‐coupled receptor for relaxin‐3	1.71	4.59E‐05	MMRRC
*Rora*	RAR‐related orphan receptor A	Nuclear hormone receptor	1.10	4.95E‐04	Wu et al.*,* (2010)
*Trh*	Thyrotropin‐releasing hormone	Hormone	1.50	1.67E‐03	Sugrue et al.*,* (2010)
*Nrp2*	Neuropilin 2	Transmembrane receptor for semaphorin 3C	0.42	4.10E‐02	Wiszniak et al.*,* (2015)

It is important to note that whilst each step of our prioritisation approach was helpful in generating a manageable list of candidates for validation, each step could have lead to us discarding useful candidates. For example, we restricted our PANTHER analysis to certain gene sub‐categories. Moreover, in some cases gene expression data are not available in the Allen Mouse Brain Atlas, or expression can be difficult to assess based on the images available. Lastly, it is important to note that there may also be many good candidate markers for which cre‐driver mouse lines do not currently exist but that could be profitably investigated following validation with immunolabelling and the future creation of cre‐driver mouse lines.

**Figure 4:**
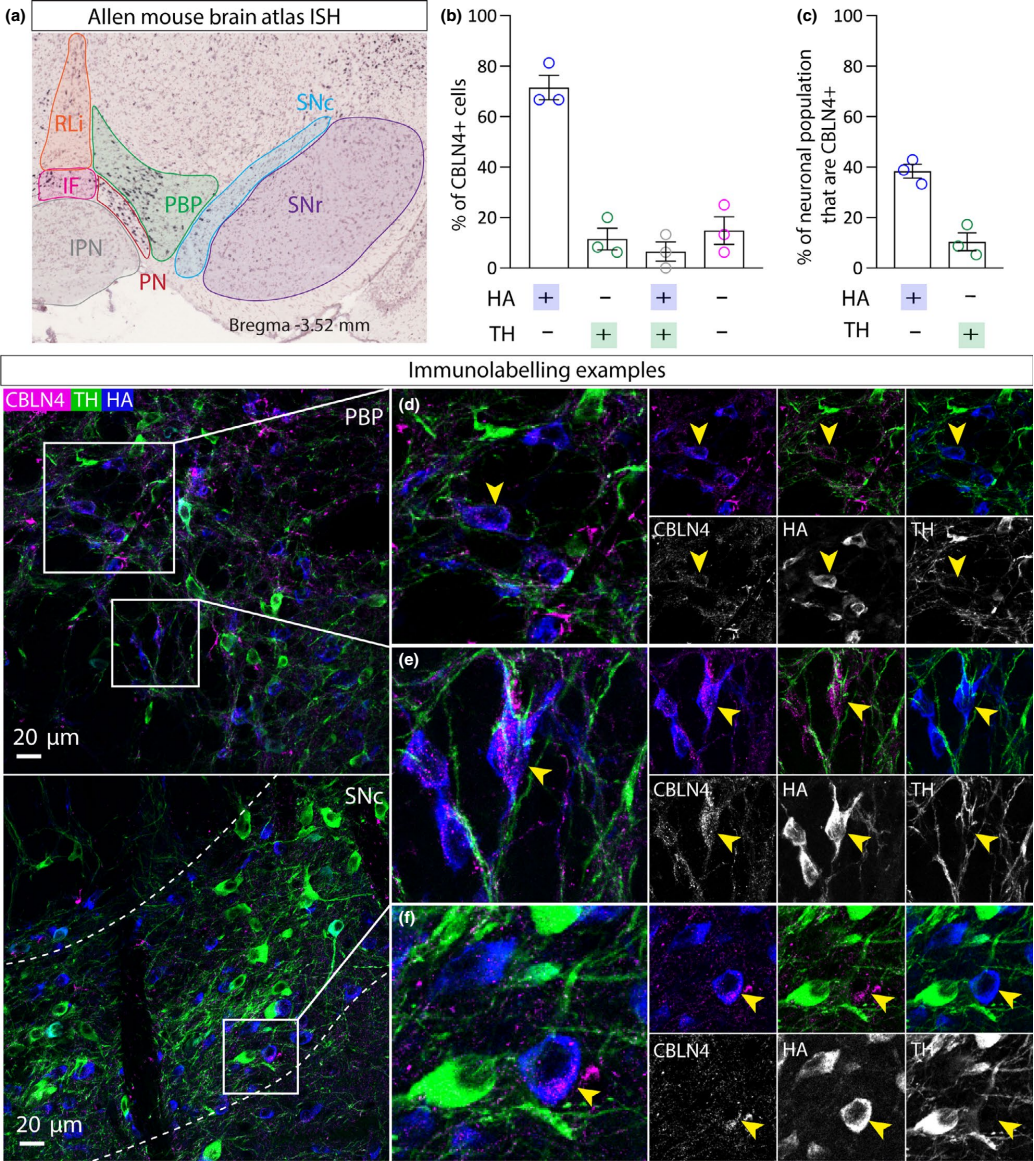
CBLN4 expression in GABA neurons in the VTA. a, Left panel shows ISH image from the Allen Mouse Brain Atlas showing mosaic expression of CBLN4 in the PN and medial PBP. b, Graph showing mean (+*SEM*) percentage of CBLN+ cells (*n* = 59 cells) that express either HA, or TH, or HA and TH, or neither. c, Graph showing mean (+*SEM*) percentage of HA+ cells (*n* = 124 cells) that express CBLN and the percentage of TH+ cells (*n* = 94 cells) that express CBLN. d, e, Representative examples of immunolabelling of CBLN4 (magenta), HA (blue) and TH (green) in the VTA of a VGATCre:RiboTag mouse, illustrating exemplar cells exhibiting CBLN4 co‐localisation with HA, but not TH (yellow arrows). f, Representative example of immunolabelling in the SNc. [Colour figure can be viewed at http://www.wileyonlinelibrary.com]

Cerebellin 4 precursor (CBLN4) belongs to a small family of glycoproteins (cerebellins) that belong to the C1q/tumour necrosis factor superfamily. The family consists of four members CBLN1‐4, with CBLN1 being the most studied prototypical family member (Chacón et al., 2015; Matsuda & Yuzaki, 2011; Wei et al., 2012). The cerebellin proteins are involved in the formation and maintenance of synapses in various brain regions (Matsuda & Yuzaki, 2011). CBLN4 binds strongly to presynaptic deleted in colorectal cancer (DDC), a transmembrane receptor that has a well‐established role in axon guidance (Wei et al., 2012). In the hippocampus, CBLN4 expression is localised to GABA interneurons where it plays a role in regulating synapses onto pyramidal neurons (Chacón et al., 2015). For this reason, it has been suggested that deficits in *Cbln4* expression may participate in the onset of Alzheimer's disease (Chacón et al., 2015). Interestingly, CBLN4 expression is restricted to the paranigral (PN) and medial parts of the parabrachial pigmented (PBP) part of the VTA, and it is not expressed in the SNC (Allen Mouse Brain Atlas; Figure 4a). We conducted triple immunolabelling for CBLN4, HA and TH in VGATCre:RiboTag mice (*n* = 3) which revealed a similar expression pattern in the PN and PBP. CBLN4 immunoreactivity appeared in a punctate‐like fashion, which has previously reported in other brain regions (Wei et al., 2012). Close examination of triple immunolabelling in the VTA revealed extensive co‐localisation between CBLN4 and HA, but not TH, showing that CBLN4 is mostly expressed in a sub‐group of GABA neurons (comprising 38% of the GABA population; Figure 4b–e). However, we did observe a small group of TH‐expressing neurons which also expressed CBLN4, consistent with single‐cell RNAseq approaches suggesting that CBLN4 may be expressed by a subset of ventromedial VTA dopamine neurons (La Manno et al., 2016). We also noted that 15% of cells expressing CBLN4 did not express either HA or TH, suggesting that CBLN4 may also be expressed in glutamate neurons or glial cells. In addition, consistent with the Allen Mouse Brain Atlas, we found that CBLN4 was generally absent from the neighbouring SNc, although where it was expressed it was typically colocalised with HA but not TH (Figure 4f).

**Figure 5:**
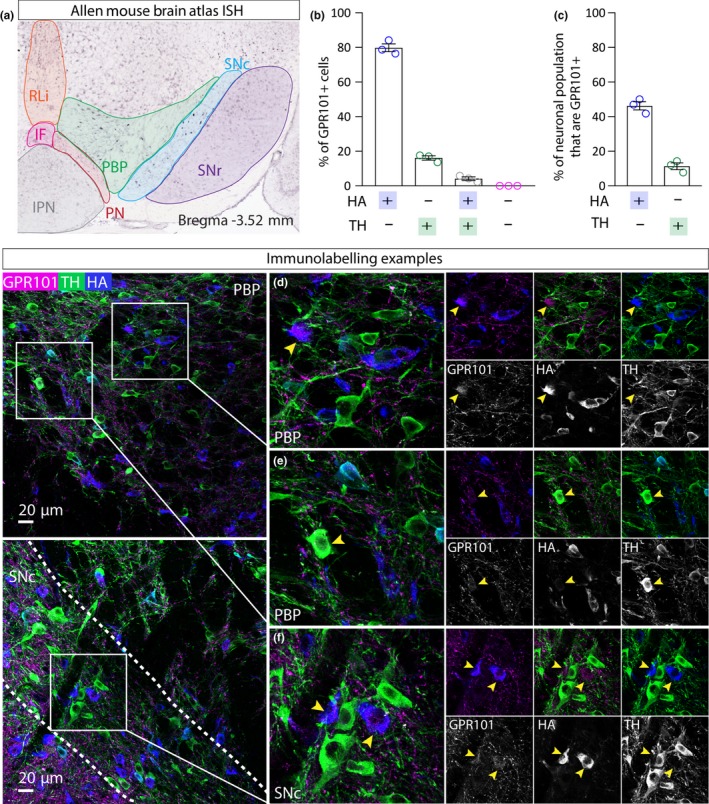
GPR101 expression in GABA neurons in the VTA. a, Left panel shows ISH image from the Allen Mouse Brain Atlas showing mosaic expression of GPR101 in the VTA and SNC. b, Graph showing mean (+*SEM*) percentage of GPR101+ cells (*n* = 230 cells) that express either HA, or TH, or HA and TH, or neither. c, Graph showing mean (+*SEM*) percentage of HA+ cells (*n* = 184) that express CBLN and the percentage of TH+ cells (*n* = 230 cells) that express GPR101. d, e, Representative examples of immunolabelling of GPR101 (magenta), HA (blue) and TH (green) in the VTA of a VGATCre:RiboTag mouse, illustrating exemplar cells exhibiting GPR101 co‐localisation with HA, but not TH (yellow arrows). f, Representative example of immunolabelling in the SNc. [Colour figure can be viewed at http://www.wileyonlinelibrary.com]

G protein‐coupled receptor 101 (GPR101) is an orphan G protein‐coupled receptor of unknown function. A role for GPR101 has been suggested in brain and pituitary development (Trivellin et al., 2016), and gene mutations have been linked to acromegaly and gigantism (Trivellin et al., 2014). In contrast to CBLN4, GPR101 is expressed throughout the VTA and SNC (Allen Mouse Brain Atlas; Figure 5a). Triple immunolabelling for GPR101, HA and TH in VGATCre:RiboTag mice (*n* = 3) revealed a similar expression pattern in the VTA and SNC. Although GPR101+ cell bodies can be resolved, immunoreactivity was more clearly seen in dendrites. Close examination of triple immunolabelling in the VTA revealed, in cases where GPR101+ cells bodies were observed, that they were mostly HA+ and TH‐ (Figure 5B–E), suggesting that GPR101 is expressed by a sub‐group of GABA neurons (46%; Figure 5c). In addition, we observed neurons expressing GPR101 and TH suggesting that GPR101 may also be expressed by a small sub‐group of dopamine neurons (11%; Figure 5c). We also observed qualitatively similar results in the SNc (Figure 5f).

**Figure 6:**
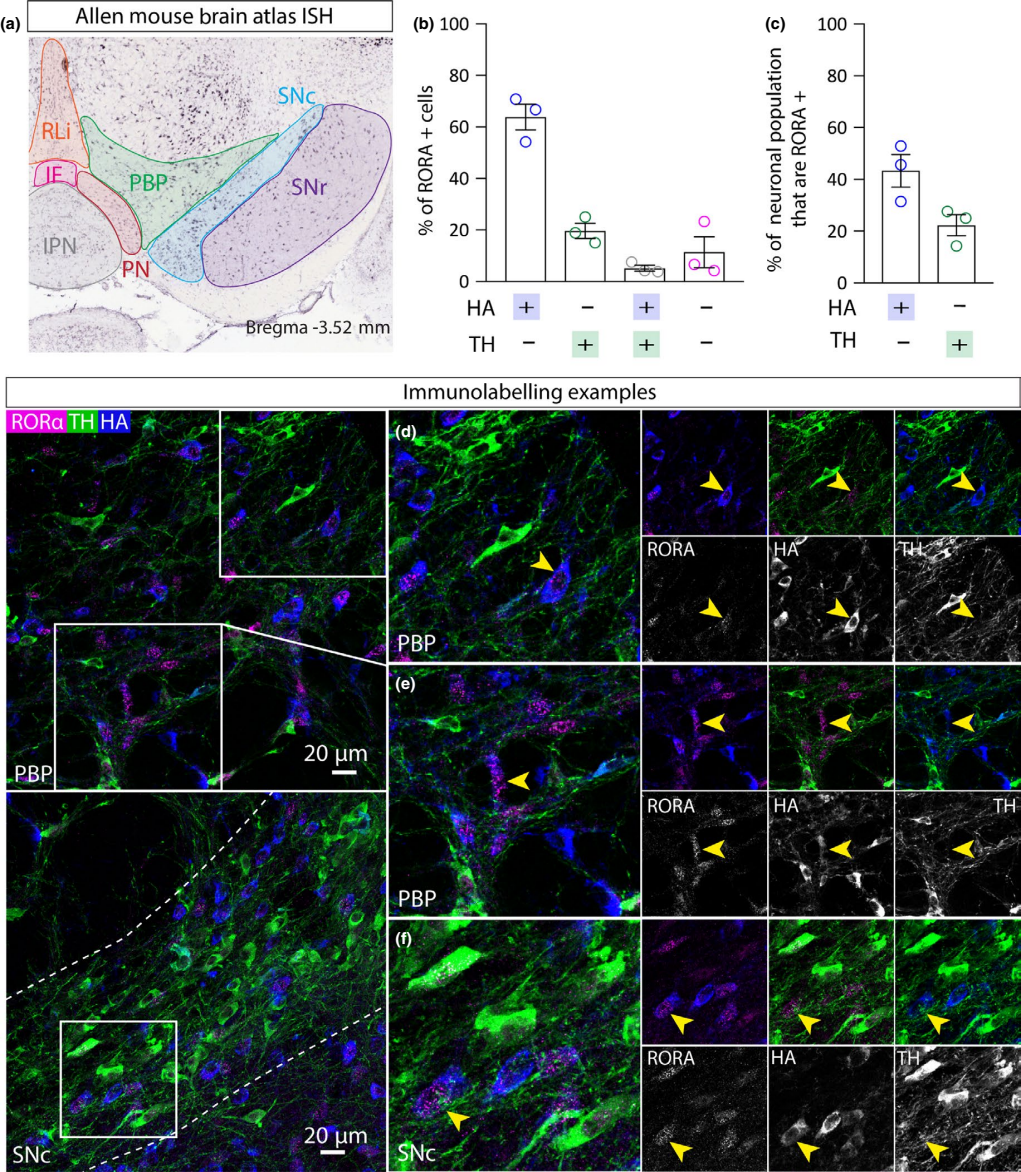
RORA expression in GABA neurons in the VTA. a, Left panel shows ISH image from the Allen Mouse Brain Atlas showing mosaic expression of RORA in the VTA and SNC. b, Graph showing mean (+SEM) percentage of RORA+ cells (*n* = 250 cells) that express either HA, or TH, or HA and TH, or neither. c, Graph showing mean (+*SEM*) percentage of HA+ cells (*n* = 367) that express RORA and the percentage of TH+ cells (*n* = 244 cells) that express RORA. d, e, Representative examples of immunolabelling of RORA (magenta), HA (blue) and TH (green) in the VTA of a VGATCre:RiboTag mouse, illustrating exemplar cells exhibiting RORA co‐localisation with HA, but not TH (yellow arrows). f, Representative example of immunolabelling in the SNc. [Colour figure can be viewed at http://www.wileyonlinelibrary.com]

RAR‐related orphan receptor A (Rora) is a member of the NR1 subfamily of nuclear hormone receptors. It is expressed in abundance in the suprachiasmatic nucleus (SCN) and is known to be necessary for normal circadian rhythms in mice (Akashi & Takumi, 2005; Ko & Takahashi, 2006; Sato et al., 2004). RORA binds to retinoic acid‐related orphan receptor response elements (ROREs) present in the promoter of *Bmal1* and important circadian clock gene (Ko & Takahashi, 2006). It is also known to be critical for the development of the cerebellum (Dussault, Fawcett, Matthyssen, Bader, & Giguère, 1998). Gene variants of RORA have been linked to autism spectrum disorder (ASD; Akashi & Takumi, 2005). RORA is expressed sparsely throughout the VTA and SNC (Allen Mouse Brain Atlas; Figure 6a). Triple immunolabelling for RORA, HA and TH in VGATCre:RiboTag mice (*n* = 3) revealed a similar expression pattern in the VTA and SNC. Close examination of triple immunolabelling in the VTA revealed a high degree of co‐expression between RORA and HA (with occasional co‐localisation with TH), suggesting that it is mostly expressed by a sub‐group of GABA neurons (43%; Figure 6b–e). We also observed qualitatively similar results in the SNc (Figure 6f).

**Figure 7:**
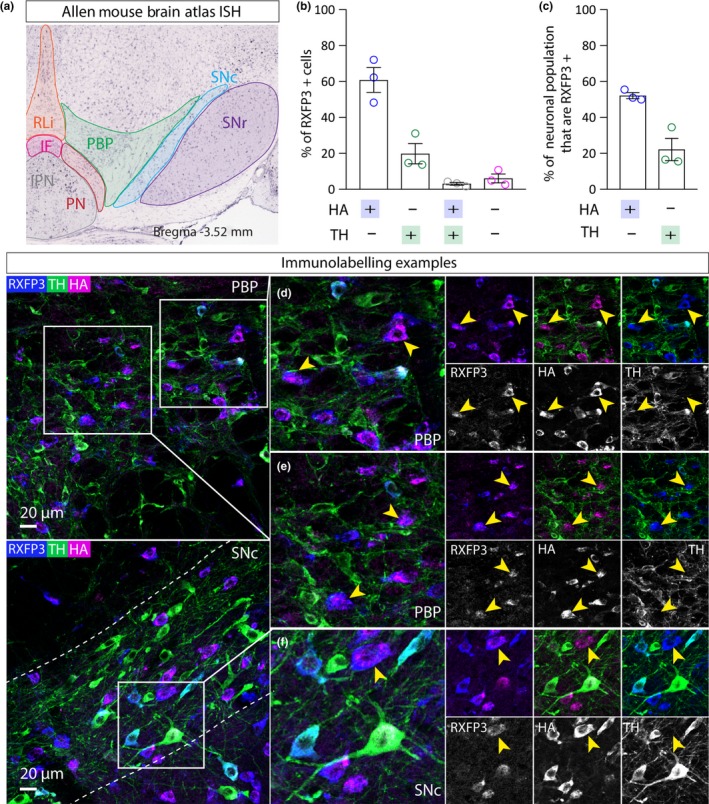
RXFP3 expression in GABA neurons in the VTA. a, Left panel shows ISH image from the Allen Mouse Brain Atlas showing mosaic expression of RORA in the VTA and SNC. b, Graph showing mean (+SEM) percentage of RXFP3+ cells (*n* = 442 cells) that express either HA, or TH, or HA and TH, or neither. c, Graph showing mean (+*SEM*) percentage of HA+ cells (*n* = 526) that express RXFP3 and the percentage of TH+ cells (*n* = 394 cells) that express RXFP3. d, e, Representative examples of immunolabelling of RXFP3 (magenta), HA (blue) and TH (green) in the VTA of a VGATCre:RiboTag mouse, illustrating exemplar cells exhibiting RXFP3 co‐localisation with HA, but not TH (yellow arrows). f, Representative example of immunolabelling in the SNc. [Colour figure can be viewed at http://www.wileyonlinelibrary.com]

Relaxin/insulin‐like family peptide receptor 3 (RXFP3) is a G protein‐coupled receptor that is selectively activated by relaxin‐3, a neuropeptide that is expressed in a small number of GABA neuron populations across the brain (Smith et al., 2010). Whilst the expression of RXFP3 and the activity of relaxin‐3 have not yet been investigated in the VTA, a small population of neurons (~350) dorsal to the SNC express relaxin‐3 (Ma, Smith, Blasiak, & Gundlach, 2017; Ma et al., 2007; Smith et al., 2010). Anatomical and pharmacological evidence suggests a role for relaxin‐3/RXFP3 signalling in motivation, anxiety and stress‐related behaviours, circadian rhythms and learning and memory (Ma et al., 2017). RXFP3 is expressed sparsely in the VTA and SNC, with most cells found in the more rostromedial regions of the VTA (Allen Mouse Brain Atlas; Figure 7a). Triple immunohistochemistry for RXFP3, HA and TH in VGATCre:RiboTag mice (*n* = 3) revealed a similarly sparse expression pattern. Close examination of triple immunolabelling in the VTA revealed extensive co‐expression between the RXFP3+ and HA+, showing that it is expressed by a sub‐group of GABA neurons (52%; Figure 7b–e). Interestingly, we also observed some co‐expression of RXFP3 and TH, suggesting that it may identify a sub‐population of dopamine. We also observed qualitatively similar results in the SNc (Figure 7f).

**Figure 8:**
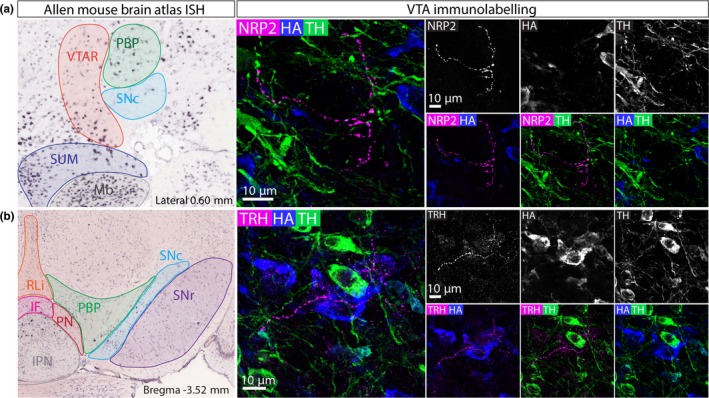
NRP2 and TRH expression in GABA neurons in the VTA. a, Left panel shows ISH image from the Allen Mouse Brain Atlas showing mosaic expression of NRP2 in the VTA and SNC (sagittal view). Right panel shows a representative example of immunolabelling of NRP, HA and TH in the VTA of a VGATCre:RiboTag mouse. NRP2 is selectively expressed in processes that do not express TH. b, Left panel shows ISH image from the Allen Mouse Brain Atlas showing mosaic expression of TRH in the VTA and SNC. Right panel shows a representative example of immunolabelling of TRH, HA and TH in the VTA of a VGATCre:RiboTag mouse. TRH is selectively expressed in processes that do not express TH. [Colour figure can be viewed at http://www.wileyonlinelibrary.com]

Neuropilin 2 (NRP2) is a transmembrane receptor that binds semaphorins SEMA3C and SEMA3F (Chen et al., 2000; Torigoe et al., 2013). NRP2 and its ligand activity have been linked to the axon guidance of various neuronal populations during development, including dopamine neurons Kolk et al., 2009;. NRP2 is expressed throughout the VTA and SNC (Allen Mouse Brain Atlas; only available in sagittal sections; Figure 8a). Triple immunolabelling for NRP2, HA and TH in VGATCre:RiboTag mice (*n *= 3) revealed NRP2+ dendrite‐like processes, sparsely distributed throughout the VTA and SNC, although they are seen in slightly higher numbers in more posterior (caudal) regions of the VTA. NRP2 expression in the VTA and SNC appears to be restricted to dendrites, consistent with NRP2 immunolabelling in the cortex (Tran et al., 2009). Because of this, it was not possible to examine co‐expression with HA (which is largely restricted to cell bodies), but all NRP2+ processes were TH‐ (Figure 8a), suggesting that NRP is expressed by a sub‐group of GABA and/or glutamate neurons.

Thyrotropin‐releasing hormone (TRH) is a neuropeptide that has an important role in the regulation of energy homeostasis, feeding behaviour, thermogenesis and autonomic regulation (Joseph‐Bravo, Jaimes‐Hoy, & Charli, 2015, 2016). TRH is expressed very sparsely in the VTA and SNC; Allen Mouse Brain Atlas; Figure 8b). Triple immunolabelling for TRH, HA and TH in VGATCre:RiboTag mice (*n* = 3) revealed sparse dendritic processes throughout the VTA but no cell bodies (Figure 8b). As for NRP+, this meant it was hard to assess co‐localisation with HA, but TRH dendrites were TH‐, suggesting that TRH is expressed by a sub‐group of GABA and/or glutamate neurons. It is potentially important to note, in this case, that TRH is expressed in the hypothalamus, and it is therefore possible that some of these processes come from neurons in that region, rather than cell bodies in the VTA.

## 4 DISCUSSION

GABA neurons in the VTA have been shown to play roles in a wide range of behaviours, including aversion, feeding suppression, associative learning, cardiovascular responses, sleep and the rewarding properties of opiates (Brown et al., 2012; Fields & Margolis, 2015; Kirouac et al., 2004; Li et al., 2019; Takata et al., 2018; Tan et al., 2012; Wakabayashi et al., 2019; van Zessen et al., 2012). Given this diversity, it seems likely that some of these effects will be mediated by functionally and anatomically distinct sub‐groups of GABA neurons. Indeed, in many other brain regions, GABA neurons (particularly interneurons) exhibit considerable physiological, anatomical and molecular diversity (e.g. Klausberger & Somogyi, 2008). However, it has been difficult to explore this diversity in the VTA in the absence of molecular markers which identify discrete sub‐groups of GABA neurons that could then be used as genetic entry points for targeted investigations (e.g. by using cre‐driver mouse lines for anatomical and functional manipulations of genetically defined cell groups). We, therefore, carried out transcriptional profiling of samples enriched for either GABA neurons or dopamine neurons. We then used the Allen Brain Atlas to identify genes that were also expressed in a mosaic fashion in the VTA. Importantly, our approach identified nNOS as a potential candidate. This provided a useful validation of the overall approach, because we and others have recently found that nNOS is expressed by a sub‐group of GABA neurons in the PBP and the SNC (Paul et al., 2018). Interestingly, these neurons do not make detectable projections outside of the VTA and SNC and may therefore be interneurons (Paul et al., 2018; Yu et al., 2019). Indeed, although it seems likely that some GABA neurons in the VTA are interneurons, it has been challenging to obtain direct evidence for this (note that although it is well established that some GABA neurons in the VTA do make local synaptic connections, the most parsimonious view has been that they are the same GABA neurons that project to regions such as the nucleus accumbens).

Having identified six candidate genes, we conducted immunolabelling to examine their expression patterns in the VTA and the degree to which they were expressed by sub‐groups of GABA neurons. CBLN4 was of particular interest because its expression was largely restricted to GABA neurons in the PN and medial PBP within the VTA. Dopamine neurons in these regions have been shown to have roles in processing aversive stimuli that are distinct from those in more lateral regions (e.g. Brischoux, Chakraborty, Brierley, & Ungless, 2009; Lammel et al., 2012). It will be interesting to see whether CBLN4‐expressing GABA neurons regulate the activity of these dopamine neurons. As was the case for the other candidates investigated, we noted small numbers of CBLN4‐expressing cells that were dopaminergic or putatively glutamatergic (and, indeed, single‐cell RNAseq results suggest that some dopamine neurons in the medial VTA may express CBLN4, La Manno et al., 2016). It may, therefore, be necessary to take a combinatorial approach to investigating these sub‐groups in the future to obtain neurochemical specificity (i.e. by using combined cre‐based (see Table 3) and flp‐based (e.g. flp‐VGAT mice) expression systems). GPR101 and RORA were also found to be expressed in a mosaic fashion in GABA neurons throughout the VTA. RXFP3 was somewhat unusual in being found mostly in rostromedial parts of the VTA. Lastly, NRP2 and TRH were sparsely expressed throughout the VTA. In both cases, expression was restricted to non‐dopaminergic dendrites, suggesting that they were GABA and/or glutamate neurons. We also observed similar expression patterns in the SNc (except for CBLN4, which is largely absent) suggesting that a more in‐depth future investigation in that region may be valuable.

Taken together, these findings present a number of promising candidates for studying sub‐groups of GABA neurons in the VTA. More broadly, the approach we took to identify mosaically expressed candidates from a regional tissue sample (in particular, the integrated use of RNAseq data with ISH image analysis from the Allen Mouse Brain Atlas) may be usefully applied to the identification of neuronal sub‐group molecular markers in other brain regions for which RNAseq data sets already exist or could be generated in a relatively straightforward manner.

## CONFLICT OF INTEREST

The authors declare no competing financial interests.

## AUTHOR CONTRIBUTION

EP, KT, and MU designed the experiments. EP performed the experiments. EP, KT, and MU analysed the data. EP and MU wrote the manuscript.

## Data Availability

Data will be made available at GEO.
