# Supplementary material for: Transcriptional profiling aligned with in situ expression image analysis reveals mosaically expressed molecular markers for GABA neuron sub‐groups in the ventral tegmental area
Source: Eur J Neurosci. 2019 Aug 16;50(11):3732–49. doi: 10.1111/ejn.14534 (PMC6972656; doi:10.1111/ejn.14534)
Supplement: Supplementary file 1 [file EJN-50-3732-s001.docx]

**Appendix**

**Table 1 – Checklist of candidate selection process**

This table contains a complete list of genes extracted from PANTHER and aligned to the data extracted from the Allen mouse brain atlas. ABA Density – refers to the % of GABAergic neurons where the value for Vgat (Slc32a1) is taken to be 100% of the population. ✓- referrers to those genes whose expression density falls within our selected range of 5-50% of the GABAergic population. Some genes fall into more than one category due to multiple experiments in the ABA having different density values. NA refers to those genes that were not included in the data extracted from the mouse brain atlas.

| **Gene Symbol** | **Log2 Fold-Change** | **P-Adj** | **Mean RPKM GABA-IP** | **Mean RPKM DA-IP** | **PANTHER** | **ABA density** | **ABA ISH** | **Cre-Driver** |
| --- | --- | --- | --- | --- | --- | --- | --- | --- |
| NAcc2 | 1.79 | 2.84E-25 | 3231.74 | 911.3267 | ✓ | ✓ |  |  |
| Penk | 2.81 | 7.18E-25 | 1796.75 | 228.77 | ✓ | <5 |  |  |
| Chga | 2.15 | 6.29E-24 | 4896.917 | 1049.323 | ✓ | >100 |  |  |
| Kcng4 | 3.32 | 4.06E-19 | 1389.357 | 106.44 | ✓ | ✓ |  |  |
| Lhx5 | 2.81 | 1.55E-18 | 318.82 | 39.03333 | ✓ | ✓ |  |  |
| KcNA6 | 1.28 | 1.80E-18 | 1464.79 | 593.5133 | ✓ | ✓ |  |  |
| **Gata3** | **2.44** | **9.03E-18** | **365.6367** | **61.13** | **✓** | **✓** | **✓** | **✓** |
| Laptm4b | 1.66 | 6.37E-17 | 1168.67 | 357.1167 | ✓ | >100 |  |  |
| Arl4c | 1.63 | 3.74E-15 | 1518.903 | 472.9867 | ✓ | <5 |  |  |
| Ranbp6 | 1.15 | 6.47E-15 | 809.07 | 362.4967 | ✓ | <5 |  |  |
| Gjd2 | 1.82 | 1.86E-14 | 192.1167 | 51.12 | ✓ | ✓ |  |  |
| Pnoc | 2.82 | 3.01E-14 | 386.61 | 43.27 | ✓ | ✓ | ✓ |  |
| Cox5a | 2.26 | 1.08E-13 | 4193.673 | 784.3333 | ✓ | >100 |  |  |
| Scn4b | 2.21 | 2.66E-13 | 2957.637 | 576.26 | ✓ | ✓ |  |  |
| **Slc32a1** | **2.55** | **3.38E-13** | **1914.253** | **274.8567** | **✓** | **100** |  |  |
| Slc25a4 | 1.57 | 1.05E-12 | 8989.233 | 2907.3 | ✓ | >100 |  |  |
| Atp5g3 | 1.94 | 1.18E-12 | 6655.607 | 1611.4 | ✓ | >100 |  |  |
| Esrrg | 2.25 | 3.15E-12 | 1354.817 | 251.96 | ✓ | ✓ |  |  |
| Cox4i1 | 1.7 | 3.48E-12 | 8413.47 | 2471.823 | ✓ | >100 |  |  |
| Cited1 | 2.53 | 3.77E-12 | 137.5733 | 19.28 | ✓ | ✓ |  |  |
| Epn3 | 1.97 | 3.85E-12 | 264.1567 | 62.79333 | ✓ | ✓ |  |  |
| Htr7 | 1.46 | 5.38E-12 | 284.9833 | 100.4 | ✓ | <5 |  |  |
| Lgi2 | 1.88 | 9.14E-12 | 2850.133 | 722.42 | ✓ | ✓ |  |  |
| Fgf9 | 1.56 | 9.49E-12 | 542.7533 | 175.7933 | ✓ | >100 |  |  |
| Esrrb | 2.65 | 1.20E-11 | 158.5267 | 19.49333 | ✓ | ✓ |  |  |
| Paqr4 | 1.56 | 1.23E-11 | 1279.427 | 414.3 | ✓ | ✓ |  |  |
| Htr2c | 1.42 | 1.32E-11 | 1269.547 | 460.8033 | ✓ | <5 |  |  |
| Atp5b | 1.54 | 2.13E-11 | 18696.36 | 6189.863 | ✓ | >100 |  |  |
| Tmem130 | 1.67 | 2.29E-11 | 6612.5 | 1980.813 | ✓ | >100 |  |  |
| Atp5a1 | 1.73 | 2.84E-11 | 16712.08 | 4761.343 | ✓ | >100 |  |  |
| Asic4 | 1.9 | 2.85E-11 | 569.2467 | 140.4267 | ✓ | NA |  |  |
| Cdc42ep2 | 1.65 | 5.84E-11 | 203.4067 | 60.59667 | ✓ | <5 |  |  |
| Dner | 2.2 | 6.87E-11 | 4871.447 | 931.2533 | ✓ | >100 |  |  |
| Vdac3 | 1.18 | 1.03E-10 | 2422.273 | 1052.277 | ✓ | >100 |  |  |
| Hcn2 | 1.13 | 2.12E-10 | 2742.597 | 1235.527 | ✓ | >100 |  |  |
| Gabra1 | 1.35 | 3.58E-10 | 4683.343 | 1789.973 | ✓ | >100 |  |  |
| Cd59a | 1.57 | 4.37E-10 | 350.3333 | 113.65 | ✓ | <5 |  |  |
| Cabp7 | 1.07 | 4.70E-10 | 1465.123 | 685.67 | ✓ | ✓ | ✓ |  |
| Nxph1 | 1.87 | 4.94E-10 | 681.9033 | 169.6567 | ✓ | ✓ | ✓ |  |
| Nrtn | 2.49 | 6.32E-10 | 137.3333 | 19.12667 | ✓ | <5 |  |  |
| Kcnh2 | 1.36 | 6.57E-10 | 1056.453 | 398.18 | ✓ | <5 |  |  |
| Gabarapl2 | 1.8 | 7.16E-10 | 3506.833 | 928.1567 | ✓ | >100 |  |  |
| **Sst** | **2.2** | **7.93E-10** | **2331.097** | **433.47** | **✓** | **✓** | **✓** | **✓** |
| Nxph4 | 2.09 | 1.13E-09 | 164.6833 | 32.59 | ✓ | ✓ |  |  |
| Atp5o | 1.72 | 1.13E-09 | 3584.743 | 1014.373 | ✓ | >100 |  |  |
| Edf1 | 1.53 | 1.21E-09 | 1683.71 | 552.6267 | ✓ | >100 |  |  |
| Atp1a3 | 1.48 | 1.23E-09 | 28744.88 | 9889.833 | ✓ | >100 |  |  |
| Atp5g1 | 1.64 | 1.35E-09 | 2301.66 | 691.9267 | ✓ | <5 |  |  |
| Atp6v0c | 1.66 | 1.39E-09 | 10728.09 | 3181.683 | ✓ | >100 |  |  |
| Slc25a11 | 1.3 | 1.43E-09 | 1535.81 | 605.8767 | ✓ | >100 |  |  |
| Sp9 | 2.44 | 1.55E-09 | 138.32 | 20.07667 | ✓ | NA |  |  |
| Timm17a | 1.52 | 2.51E-09 | 1041.037 | 344.2733 | ✓ | <5% |  |  |
| KcNAb2 | 1.04 | 2.81E-09 | 4554.973 | 2185.977 | ✓ | ✓ |  |  |
| Cand1 | 0.89 | 3.30E-09 | 1747.043 | 936.9867 | ✓ | >100 |  |  |
| Glra1 | 2.59 | 3.39E-09 | 459.71 | 57.52667 | ✓ | ✓ |  |  |
| Rcan2 | 1.48 | 4.43E-09 | 4398.79 | 1504.743 | ✓ | >100 |  |  |
| Rom1 | 1.5 | 5.88E-09 | 154.58 | 52.08 | ✓ | ✓ |  |  |
| Lrrc38 | 2.37 | 5.88E-09 | 84.26333 | 12.63 | ✓ | <5% |  |  |
| B3galt5 | 0.92 | 6.69E-09 | 686.4667 | 358.4333 | ✓ | ✓ |  |  |
| Kcnk3 | 1.17 | 6.87E-09 | 539.1467 | 233.5933 | ✓ | NA |  |  |
| Strap | 1.07 | 7.16E-09 | 1677.287 | 785.7 | ✓ | <5/>100 |  |  |
| Slc35g2 | 1.72 | 7.60E-09 | 462.16 | 129.8433 | ✓ | NA |  |  |
| Kcnip1 | 1.47 | 8.31E-09 | 482.15 | 165.0667 | ✓ | ✓ |  |  |
| Ctnnbip1 | 1.12 | 9.05E-09 | 782.7133 | 351.03 | ✓ | ✓ |  |  |
| Vdac2 | 0.97 | 1.01E-08 | 2453.1 | 1235.567 | ✓ | ✓ |  |  |
| Atp5j2 | 1.9 | 1.11E-08 | 3096.203 | 743.7867 | ✓ | ✓ |  |  |
| Sall2 | 0.96 | 1.34E-08 | 918.24 | 465.3133 | ✓ | ✓ |  |  |
| Park7 | 1.55 | 1.57E-08 | 2516.723 | 807.8633 | ✓ | >100 |  |  |
| Wbp5 | 1.76 | 1.78E-08 | 1506.433 | 406.15 | ✓ | >100 |  |  |
| BC031181 | 1.43 | 1.90E-08 | 1549.63 | 547.4567 | ✓ | ✓ |  |  |
| Ndrg4 | 1.19 | 1.90E-08 | 28873.88 | 12361.5 | ✓ | >100 |  |  |
| Diras1 | 1.66 | 2.33E-08 | 3018.373 | 883.7033 | ✓ | <5/>100 |  |  |
| Slc1a6 | 2.08 | 2.50E-08 | 512.3767 | 103.5867 | ✓ | ✓ |  |  |
| Hpcal1 | 1.69 | 2.76E-08 | 1352.763 | 385.6033 | ✓ | ✓ |  |  |
| Atp5e | 1.93 | 2.87E-08 | 1497.373 | 346.45 | ✓ | <5 |  |  |
| Cib2 | 1.47 | 3.19E-08 | 611.9167 | 209.97 | ✓ | <5 |  |  |
| Vdac1 | 1.17 | 3.29E-08 | 6534.077 | 2829.883 | ✓ | >100 |  |  |
| Gnl1 | 1.02 | 3.49E-08 | 2654.463 | 1282.653 | ✓ | ✓ |  |  |
| Atp6v1h | 1.04 | 3.93E-08 | 2108.567 | 1010.333 | ✓ | >100 |  |  |
| Irx3 | 1.92 | 4.34E-08 | 121.7167 | 27.62667 | ✓ | <5 |  |  |
| Akt1 | 0.81 | 4.53E-08 | 2237.667 | 1262.777 | ✓ | >100 |  |  |
| Spryd3 | 1.27 | 4.65E-08 | 2634.163 | 1056.483 | ✓ | >100 |  |  |
| Tal1 | 1.63 | 4.74E-08 | 351.97 | 105.65 | ✓ | <5 |  |  |
| Gpr62 | 1.65 | 4.84E-08 | 198.0867 | 57.5 | ✓ | <5 |  |  |
| Ap2m1 | 0.94 | 5.04E-08 | 6138.733 | 3151.987 | ✓ | >100 |  |  |
| Fgf18 | 2 | 6.27E-08 | 60.66 | 12.98333 | ✓ | ✓ |  |  |
| Mtch1 | 1.15 | 6.68E-08 | 5388.947 | 2364.867 | ✓ | >100 |  |  |
| Scn1b | 1.18 | 6.86E-08 | 4537.47 | 1946.817 | ✓ | >100 |  |  |
| Vps45 | 0.98 | 9.58E-08 | 418.0267 | 207.5967 | ✓ | NA |  |  |
| Fkbp2 | 1.38 | 1.13E-07 | 1623.637 | 598.25 | ✓ | <5 |  |  |
| Tac1 | 1.54 | 1.17E-07 | 392.32 | 126.58 | ✓ | <5 |  |  |
| Slc25a5 | 1.44 | 1.22E-07 | 2508.937 | 877.3267 | ✓ | >100 |  |  |
| Slc41a3 | 1.84 | 1.33E-07 | 203.89 | 49.90667 | ✓ | ✓ |  |  |
| Cacng5 | 1.47 | 1.46E-07 | 208.45 | 70.36 | ✓ | ✓ |  |  |
| Pink1 | 1.01 | 1.49E-07 | 3796.13 | 1844.77 | ✓ | >100 |  |  |
| Arl3 | 1.26 | 1.59E-07 | 1120.197 | 449.7633 | ✓ | <5/>100 |  |  |
| Tceal1 | 1.61 | 1.63E-07 | 619.2367 | 186.3733 | ✓ | >100 |  |  |
| Srp9 | 1.34 | 2.05E-07 | 1469.057 | 553.12 | ✓ | <5 |  |  |
| Atp6v1a | 1.11 | 2.17E-07 | 6673.747 | 3011.87 | ✓ | >100 |  |  |
| Zc3h15 | 0.97 | 2.18E-07 | 1838.573 | 924.09 | ✓ | >100 |  |  |
| GNAs | 1.34 | 2.65E-07 | 20626.89 | 7791.087 | ✓ | >100 |  |  |
| Crhbp | 1.52 | 3.04E-07 | 206.07 | 66.22 | ✓ | ✓ | ✓ |  |
| Hgs | 0.89 | 3.19E-07 | 956.4967 | 509.55 | ✓ | <5 |  |  |
| Scn2b | 1.07 | 3.63E-07 | 3641.967 | 1695.46 | ✓ | >100 |  |  |
| Nell1 | 1.57 | 4.01E-07 | 962.4 | 301.4933 | ✓ | <5 |  |  |
| PitpNA | 1.02 | 4.06E-07 | 4970.797 | 2406.947 | ✓ | >100 |  |  |
| Atp5d | 1.34 | 4.15E-07 | 2996.69 | 1126.387 | ✓ | >100 |  |  |
| Map1lc3a | 1.45 | 4.40E-07 | 2539.693 | 874.2067 | ✓ | ✓ |  |  |
| Slc6a1 | 0.67 | 4.56E-07 | 3715.207 | 2325.19 | ✓ | ✓ |  |  |
| Cdip1 | 0.95 | 5.16E-07 | 3498.31 | 1777.973 | ✓ | NA |  |  |
| Eef1e1 | 1.41 | 5.65E-07 | 461.84 | 162.8133 | ✓ | <5 |  |  |
| Tceb2 | 1.54 | 6.08E-07 | 1577.92 | 504.0033 | ✓ | >100 |  |  |
| Nfe2l1 | 0.87 | 8.16E-07 | 4413.38 | 2381.94 | ✓ | >100 |  |  |
| Fgf1 | 1.29 | 8.59E-07 | 1267.853 | 493.4033 | ✓ | >100 |  |  |
| Acp1 | 1.07 | 8.88E-07 | 896.7633 | 417.82 | ✓ | ✓ |  |  |
| Lrrc49 | 0.89 | 1.04E-06 | 1231.783 | 652.9367 | ✓ | >100 |  |  |
| ChrNA2 | 2.28 | 1.19E-06 | 50.75333 | 7.256667 | ✓ | ✓ |  |  |
| Nr2f2 | 1.15 | 1.24E-06 | 607.7667 | 265.5767 | ✓ | ✓ |  |  |
| Tomm7 | 1.57 | 1.29E-06 | 815.01 | 249.9167 | ✓ | NA |  |  |
| Timm22 | 1.32 | 1.33E-06 | 457.6533 | 174.0367 | ✓ | >100 |  |  |
| Kcnk1 | 1.03 | 1.53E-06 | 879.43 | 418.1467 | ✓ | <5 |  |  |
| Fzd1 | 1.09 | 1.56E-06 | 210.48 | 95.46 | ✓ | ✓ |  |  |
| Timm10 | 1.52 | 1.59E-06 | 313.2467 | 99.81667 | ✓ | ✓ |  |  |
| Clpp | 0.91 | 1.67E-06 | 726.94 | 380.6033 | ✓ | NA |  |  |
| Zfp385a | 1.33 | 1.67E-06 | 1533.727 | 580.79 | ✓ | >100 |  |  |
| Leprotl1 | 1.18 | 1.68E-06 | 1050.467 | 445.77 | ✓ | >100 |  |  |
| Chrm2 | 1.48 | 1.73E-06 | 325.0467 | 108.2167 | ✓ | ✓ |  |  |
| Lrrn2 | 1.14 | 1.73E-06 | 1247.713 | 547.2733 | ✓ | <5/>100 |  |  |
| Gabrg2 | 0.92 | 1.82E-06 | 2167.853 | 1131.097 | ✓ | >100 |  |  |
| Nkiras1 | 1.03 | 1.85E-06 | 1891.387 | 905.7367 | ✓ | ✓ |  |  |
| Kcnc1 | 1.04 | 1.87E-06 | 3686.31 | 1756.373 | ✓ | >100 |  |  |
| Irx1 | 1.33 | 1.96E-06 | 104.5567 | 38.53333 | ✓ | ✓ | ✓ |  |
| Zfp521 | 1.45 | 1.97E-06 | 341.6333 | 115.4867 | ✓ | ✓ |  |  |
| Ddt | 1.68 | 1.99E-06 | 426.1567 | 118.0633 | ✓ | ✓ |  |  |
| Relt | 1.02 | 2.00E-06 | 338.6033 | 163.5467 | ✓ | ✓ |  |  |
| Slc25a33 | 1.19 | 2.10E-06 | 718.0433 | 303.9233 | ✓ | <5 |  |  |
| Nenf | 1.55 | 2.27E-06 | 669.8533 | 209.4033 | ✓ | ✓ | ✓ |  |
| CntNAp2 | 1.29 | 2.32E-06 | 856.9733 | 334.55 | ✓ | >100 |  |  |
| Sfrp2 | 2.29 | 2.35E-06 | 67.23 | 8.67 | ✓ | ✓ |  |  |
| Tspan3 | 1.32 | 2.38E-06 | 3959.87 | 1503.787 | ✓ | >100 |  |  |
| Tomm40 | 0.72 | 2.49E-06 | 999.0767 | 600.76 | ✓ | <5 |  |  |
| Emc6 | 1.2 | 2.51E-06 | 526.0533 | 218.27 | ✓ | NA |  |  |
| KcNA1 | 1.22 | 2.57E-06 | 2886.007 | 1193.697 | ✓ | <5 |  |  |
| Bag3 | 1.22 | 2.73E-06 | 232.7433 | 94.52667 | ✓ | ✓ |  |  |
| Clec2l | 1.09 | 2.75E-06 | 998.82 | 455.4633 | ✓ | NA |  |  |
| Rcan3 | 1.12 | 2.77E-06 | 920.5133 | 411.1733 | ✓ | ✓ |  |  |
| GNAl | 1 | 2.78E-06 | 1889.363 | 922.7533 | ✓ | ✓ |  |  |
| Hbegf | 1.04 | 3.10E-06 | 172.3633 | 82.69667 | ✓ | <5 |  |  |
| Nkx6-1 | 2.17 | 3.18E-06 | 37.62 | 5.993333 | ✓ | ✓ |  |  |
| Bcap31 | 1.03 | 3.23E-06 | 805.65 | 385.5267 | ✓ | >100 |  |  |
| Iltifb | 2.68 | 3.32E-06 | 67.34333 | 5.076667 | ✓ | NA |  |  |
| Prelid1 | 1.16 | 3.38E-06 | 1476.883 | 635.3233 | ✓ | >100 |  |  |
| Spcs1 | 1.33 | 3.39E-06 | 1104.093 | 415.1767 | ✓ | >100 |  |  |
| Slc39a6 | 0.87 | 3.46E-06 | 640.35 | 344.1033 | ✓ | <5 |  |  |
| Cited2 | 1.26 | 3.76E-06 | 533.2567 | 212.2933 | ✓ | <5/>100 |  |  |
| Smyd2 | 0.93 | 4.06E-06 | 1194.53 | 613.3133 | ✓ | >100 |  |  |
| Lhx1 | 2.09 | 4.23E-06 | 221.4 | 40.24667 | ✓ | ✓ |  |  |
| **Gpr101** | **1.24** | **4.25E-06** | **188.53** | **76.74** | **✓** | **✓** | **✓** | **✓** |
| Cerk | 1.09 | 4.36E-06 | 1784.507 | 811.5367 | ✓ | ✓ |  |  |
| Nectin2 | 1.31 | 4.41E-06 | 164.9533 | 61.85 | ✓ | NA |  |  |
| Asph | 1.19 | 4.52E-06 | 1538.113 | 650.4233 | ✓ | ✓ |  |  |
| Thy1 | 1.36 | 4.52E-06 | 6444.94 | 2361.82 | ✓ | >100 |  |  |
| Gng3 | 1.31 | 4.58E-06 | 2507.127 | 958.01 | ✓ | >100 |  |  |
| Cadm4 | 1.28 | 4.78E-06 | 3546.777 | 1388.463 | ✓ | >100 |  |  |
| Slc25a25 | 0.9 | 4.83E-06 | 1287.3 | 679.1767 | ✓ | <5 |  |  |
| Dach1 | 1.34 | 4.86E-06 | 153.8433 | 57.37 | ✓ | ✓ |  |  |
| Uqcr10 | 1.34 | 4.91E-06 | 1377.41 | 513.97 | ✓ | <5 |  |  |
| Slmo2 | 1.16 | 4.93E-06 | 762.94 | 327.32 | ✓ | ✓ |  |  |
| Ctbp1 | 0.87 | 5.11E-06 | 2779.237 | 1493.28 | ✓ | >100 |  |  |
| Pax2 | 2.64 | 5.23E-06 | 54.68333 | 3.886667 | ✓ | <5 |  |  |
| Scg2 | 1.54 | 5.79E-06 | 7920.59 | 2481.303 | ✓ | >100 |  |  |
| Atg101 | 1.14 | 6.44E-06 | 367.67 | 159.59 | ✓ | NA |  |  |
| Mapk8ip2 | 0.79 | 6.64E-06 | 3794.153 | 2177.583 | ✓ | >100 |  |  |
| Znhit2 | 1.45 | 6.66E-06 | 319.8733 | 107.14 | ✓ | <5 |  |  |
| Chgb | 1.25 | 6.94E-06 | 5116.217 | 2054.723 | ✓ | >100 |  |  |
| Hap1 | 0.91 | 7.08E-06 | 4457.197 | 2340.453 | ✓ | >100 |  |  |
| Tceal3 | 1.2 | 7.42E-06 | 1337.217 | 555.2367 | ✓ | NA |  |  |
| Otp | 2.55 | 7.46E-06 | 41.39667 | 3.24 | ✓ | ✓ |  |  |
| Atp6v1c1 | 1.08 | 7.53E-06 | 2846.98 | 1310.343 | ✓ | <5/>100 |  |  |
| Slc27a4 | 0.72 | 7.60E-06 | 1475.85 | 887.3567 | ✓ | >100 |  |  |
| Gprc5b | 0.92 | 7.75E-06 | 1869.51 | 965.48 | ✓ | >100 |  |  |
| Btbd11 | 1.1 | 7.95E-06 | 698.3933 | 317.52 | ✓ | <5 |  |  |
| Colq | 1.84 | 8.09E-06 | 62.17 | 14.92667 | ✓ | <5 |  |  |
| Grid2 | 2.12 | 8.11E-06 | 416.38 | 71.31333 | ✓ | ✓ |  |  |
| Snw1 | 0.73 | 8.41E-06 | 801.76 | 478.49 | ✓ | ✓ |  |  |
| Srprb | 1 | 9.15E-06 | 441.48 | 215.34 | ✓ | ✓ |  |  |
| Psap | 0.97 | 9.71E-06 | 19073.8 | 9504.007 | ✓ | >100 |  |  |
| Cox7a2l | 1.09 | 9.71E-06 | 1533.68 | 699.64 | ✓ | >100 |  |  |
| NAca | 1.07 | 9.96E-06 | 2137.707 | 983.9333 | ✓ | >100 |  |  |
| Pepd | 0.94 | 1.05E-05 | 426.6633 | 217.26 | ✓ | <5 |  |  |
| Pax8 | 2.38 | 1.11E-05 | 32.95 | 3.44 | ✓ | <5 |  |  |
| Cacng2 | 1.51 | 1.33E-05 | 2555.06 | 816.9867 | ✓ | ✓ |  |  |
| **Cbln4** | **1.28** | **1.42E-05** | **339.7667** | **131.8633** | **✓** | **✓** | **✓** | **✓** |
| Lrrc4b | 0.85 | 1.52E-05 | 1687.767 | 920.4467 | ✓ | >100 |  |  |
| Glrb | 0.97 | 1.61E-05 | 2723.977 | 1357.263 | ✓ | >100 |  |  |
| Scg5 | 1.15 | 1.63E-05 | 2101.147 | 911.8933 | ✓ | >100 |  |  |
| Slc38a1 | 1.07 | 1.70E-05 | 3379.11 | 1561.937 | ✓ | >100 |  |  |
| Akr1a1 | 1.19 | 1.70E-05 | 4543.883 | 1910.65 | ✓ | >100 |  |  |
| Psmd14 | 0.8 | 1.70E-05 | 972.7867 | 550.4533 | ✓ | >100 |  |  |
| Ngb | 1.38 | 1.73E-05 | 225.2767 | 80.41667 | ✓ | ✓ | ✓ |  |
| Hoxb5 | 2.28 | 1.81E-05 | 22.59667 | 2.336667 | ✓ | ✓ |  |  |
| Kcnmb2 | 1.58 | 1.81E-05 | 94.18 | 27.83333 | ✓ | <5 |  |  |
| Pgrmc2 | 0.91 | 1.84E-05 | 1209.97 | 631.37 | ✓ | <5 |  |  |
| Necap1 | 1.15 | 2.04E-05 | 2958.043 | 1280.097 | ✓ | ✓ |  |  |
| Htr3a | 1.46 | 2.17E-05 | 93.14667 | 30.74333 | ✓ | <5 |  |  |
| Nova1 | 0.97 | 2.20E-05 | 1816.893 | 908.99 | ✓ | <5/>100 |  |  |
| Aes | 0.98 | 2.20E-05 | 4328.98 | 2139.803 | ✓ | >100 |  |  |
| Lmo1 | 1.15 | 2.26E-05 | 181.6033 | 78.05333 | ✓ | <5 |  |  |
| Gal | 2.12 | 2.45E-05 | 33.43333 | 5.063333 | ✓ | <5 |  |  |
| Gpr149 | 1.68 | 2.49E-05 | 48.46333 | 13.17667 | ✓ | <5 |  |  |
| Bsg | 1.12 | 2.51E-05 | 6583.403 | 2907.047 | ✓ | >100 |  |  |
| Bag5 | 1.32 | 2.61E-05 | 644.6333 | 241.16 | ✓ | ✓ |  |  |
| Cltc | 1.02 | 2.64E-05 | 7775.373 | 3737.97 | ✓ | >100 |  |  |
| Chrnb2 | 0.68 | 2.79E-05 | 1083.363 | 670.3367 | ✓ | ✓ |  |  |
| Hoxa5 | 2.47 | 2.90E-05 | 14.18333 | 0.883333 | ✓ | >100 |  |  |
| Taf7 | 1.23 | 2.94E-05 | 140.5267 | 56.36333 | ✓ | ✓ |  |  |
| Caln1 | 1.25 | 3.04E-05 | 655.6133 | 261.8833 | ✓ | <5 |  |  |
| Snd1 | 0.68 | 3.05E-05 | 950.0433 | 588.1633 | ✓ | >100 |  |  |
| Calca | 1.87 | 3.06E-05 | 63.31333 | 13.29333 | ✓ | <5 |  |  |
| Atp9a | 0.7 | 3.18E-05 | 3789.78 | 2309.207 | ✓ | ✓ |  |  |
| Panx2 | 0.6 | 3.21E-05 | 1658.003 | 1085.027 | ✓ | ✓ |  |  |
| Zbtb4 | 0.55 | 3.39E-05 | 1783.443 | 1212.36 | ✓ | <5 |  |  |
| Slco3a1 | 0.8 | 3.51E-05 | 601.0267 | 341.35 | ✓ | <5/<5 |  |  |
| Atp6v1b2 | 1.26 | 3.95E-05 | 11193.88 | 4421.313 | ✓ | >100 |  |  |
| Hmgb3 | 1.28 | 3.97E-05 | 305.1033 | 116.6367 | ✓ | NA |  |  |
| Cplx1 | 1.35 | 4.52E-05 | 15033.27 | 5501.977 | ✓ | >100 |  |  |
| **Rxfp3** | **1.71** | **4.59E-05** | **82.16333** | **21.23667** | **✓** | **✓** | **✓** | **✓** |
| Btf3 | 1.16 | 4.68E-05 | 1527.66 | 653.36 | ✓ | ✓ |  |  |
| Gnb5 | 0.82 | 4.69E-05 | 1492.13 | 829.49 | ✓ | ✓ |  |  |
| Eif3h | 0.92 | 4.73E-05 | 1038.55 | 534.65 | ✓ | >100 |  |  |
| Sox14 | 1.98 | 4.76E-05 | 70.85 | 13 | ✓ | ✓ | ✓ |  |
| Gpr176 | 1.04 | 4.93E-05 | 197.9633 | 93.30667 | ✓ | ✓ |  |  |
| Ramp3 | 1.52 | 5.00E-05 | 235.15 | 72.57 | ✓ | <5 |  |  |
| CtnNA2 | 0.95 | 5.15E-05 | 1276.057 | 643.28 | ✓ | ✓ |  |  |
| Bcl2l1 | 1 | 5.15E-05 | 1476.957 | 716.1333 | ✓ | >100 |  |  |
| Arf6 | 0.91 | 5.21E-05 | 703.47 | 364.58 | ✓ | ✓ |  |  |
| Pam16 | 0.91 | 5.38E-05 | 330.6133 | 171.5067 | ✓ | <5 |  |  |
| Ik | 0.93 | 5.43E-05 | 1972.033 | 1014.18 | ✓ | >100 |  |  |
| Disp2 | 0.59 | 5.60E-05 | 4852.68 | 3208.393 | ✓ | >100 |  |  |
| 0610009B22Rik | 1.08 | 5.72E-05 | 218.84 | 98.33 | ✓ | <5 |  |  |
| Eef1g | 0.86 | 5.78E-05 | 3423.557 | 1857.813 | ✓ | >100 |  |  |
| Alpk2 | 2.46 | 6.29E-05 | 48.64333 | 3.29 | ✓ | <5 |  |  |
| Timm8b | 1.23 | 6.47E-05 | 778.6867 | 311.3033 | ✓ | <5 |  |  |
| Tspan9 | 0.94 | 6.56E-05 | 641.6967 | 325.4167 | ✓ | ✓ | ✓ |  |
| Gsto1 | 1.07 | 6.69E-05 | 325.92 | 148.0367 | ✓ | ✓ | ✓ |  |
| Snx3 | 1.21 | 6.88E-05 | 1684.897 | 689.5133 | ✓ | >100 |  |  |
| Fxyd6 | 1.33 | 7.00E-05 | 1755.023 | 645.71 | ✓ | >100 |  |  |
| Vmp1 | 0.75 | 7.04E-05 | 923.6567 | 542.3367 | ✓ | NA |  |  |
| Pax7 | 1.81 | 7.31E-05 | 124.3933 | 27.73667 | ✓ | ✓ |  |  |
| Sik3 | 0.55 | 7.47E-05 | 1458.633 | 992.9033 | ✓ | <5 |  |  |
| Impad1 | 1.01 | 7.61E-05 | 1848.75 | 889.7733 | ✓ | <5 |  |  |
| Gbx2 | 1.54 | 9.13E-05 | 32.96333 | 9.763333 | ✓ | <5 |  |  |
| Reep1 | 0.78 | 9.40E-05 | 1601.527 | 918.1867 | ✓ | >100 |  |  |
| Morf4l1 | 0.91 | 9.67E-05 | 5902.477 | 3069.507 | ✓ | >100 |  |  |
| Paqr9 | 1.08 | 9.93E-05 | 180.81 | 80.47333 | ✓ | <5 |  |  |
| Megf11 | 1.35 | 1.01E-04 | 433.2133 | 156.28 | ✓ | ✓ | ✓ |  |
| Gne | 0.76 | 1.02E-04 | 453.86 | 263.2 | ✓ | ✓ |  |  |
| Kcnc4 | 0.86 | 1.04E-04 | 1002.367 | 539.33 | ✓ | ✓ | ✓ |  |
| Khdrbs1 | 0.69 | 1.06E-04 | 1461.263 | 895.4133 | ✓ | <5 |  |  |
| Timm17b | 1.07 | 1.08E-04 | 277.39 | 126.1333 | ✓ | <5 |  |  |
| Tfam | 0.78 | 1.10E-04 | 556.86 | 319.0967 | ✓ | <5 |  |  |
| Cuta | 1.14 | 1.22E-04 | 703.3333 | 302.54 | ✓ | <5 |  |  |
| Fbxo45 | 0.74 | 1.24E-04 | 842.4567 | 495.66 | ✓ | ✓ |  |  |
| Tceal6 | 1.31 | 1.25E-04 | 838.9233 | 311.0567 | ✓ | >100 |  |  |
| Pex14 | 1.03 | 1.27E-04 | 524.7767 | 246.7467 | ✓ | ✓ | ✓ |  |
| Atg9b | 0.88 | 1.27E-04 | 134.04 | 71.11333 | ✓ | <5 |  |  |
| Gabarapl1 | 1.07 | 1.30E-04 | 6984.357 | 3204.983 | ✓ | >100 |  |  |
| Mcfd2 | 0.97 | 1.30E-04 | 897.2667 | 443.8433 | ✓ | NA |  |  |
| Map1lc3b | 0.95 | 1.33E-04 | 2240.5 | 1127.513 | ✓ | >100 |  |  |
| Dusp10 | 1.41 | 1.35E-04 | 156.53 | 52.71333 | ✓ | ✓ |  |  |
| Slc35e4 | 0.96 | 1.35E-04 | 294.6433 | 145.5433 | ✓ | NA |  |  |
| Bnip3 | 1.01 | 1.35E-04 | 1233.073 | 593.3567 | ✓ | >100 |  |  |
| Sfrp1 | 1.52 | 1.41E-04 | 140.29 | 42.04 | ✓ | ✓ |  |  |
| Uncx | 1.33 | 1.44E-04 | 85.23667 | 31.48333 | ✓ | ✓ |  |  |
| Arhgdig | 1.01 | 1.53E-04 | 841.82 | 402.8067 | ✓ | >100 |  |  |
| Smad9 | 1.08 | 1.59E-04 | 314.5167 | 141.8967 | ✓ | <5 |  |  |
| Cdipt | 0.68 | 1.63E-04 | 1183.453 | 728.5567 | ✓ | >100 |  |  |
| Gabrb2 | 1.11 | 1.66E-04 | 2918.663 | 1288.993 | ✓ | ✓ | ✓ |  |
| Gdf11 | 0.92 | 1.66E-04 | 307.6333 | 157.8633 | ✓ | ✓ |  |  |
| Edil3 | 1.02 | 1.68E-04 | 1479.59 | 703.5467 | ✓ | ✓ |  |  |
| Rhd | 2.32 | 1.68E-04 | 11.21667 | 0.823333 | ✓ | <5 |  |  |
| Atp6v1f | 1.2 | 1.69E-04 | 1224.943 | 498.81 | ✓ | NA |  |  |
| B3gnt5 | 2.03 | 1.71E-04 | 48.46333 | 7.896667 | ✓ | <5 |  |  |
| Cbln1 | 1.58 | 1.74E-04 | 196.9833 | 55.74333 | ✓ | >100 |  |  |
| Cops8 | 0.75 | 1.80E-04 | 1130.863 | 663.8067 | ✓ | <5 |  |  |
| Gdi2 | 0.69 | 1.81E-04 | 2364.52 | 1445.08 | ✓ | <5/>100 |  |  |
| Cckar | 2.18 | 1.82E-04 | 24.53333 | 2.65 | ✓ | ✓ | ✓ |  |
| Slc25a12 | 0.75 | 1.91E-04 | 2217.16 | 1296.007 | ✓ | >100 |  |  |
| C1d | 1.11 | 1.95E-04 | 686.4033 | 303.3833 | ✓ | ✓ |  |  |
| Panx1 | 1 | 1.96E-04 | 221.53 | 106.43 | ✓ | ✓ | ✓ |  |
| Pitx2 | 1.48 | 1.96E-04 | 77.17 | 24.45 | ✓ | <5 |  |  |
| Jund | 1.3 | 1.98E-04 | 2244.913 | 838.9933 | ✓ | >100 |  |  |
| Cnr1 | 0.66 | 2.00E-04 | 1286.257 | 807.8967 | ✓ | <5>100 |  |  |
| Hdgf | 0.64 | 2.04E-04 | 2251.333 | 1430.737 | ✓ | >100 |  |  |
| Erh | 1.16 | 2.07E-04 | 758.85 | 318.45 | ✓ | ✓ |  |  |
| Pqbp1 | 1 | 2.08E-04 | 808.69 | 388.0933 | ✓ | ✓ |  |  |
| Atp6v0d1 | 0.96 | 0.000218 | 2391.237 | 1195.013 | ✓ | NA |  |  |
| Nrxn3 | 1.26 | 2.08E-04 | 4975.547 | 1928.163 | ✓ | >100 |  |  |
| Dpp6 | 0.66 | 2.19E-04 | 2533.46 | 1587.083 | ✓ | ✓ |  |  |
| Asic2 | 0.71 | 2.20E-04 | 629.2033 | 380.73 | ✓ | NA |  |  |
| Inhbb | 1.16 | 2.36E-04 | 142.3667 | 59.46 | ✓ | <5 |  |  |
| KcNAb1 | 0.96 | 2.39E-04 | 884.7233 | 440.2667 | ✓ | ✓ |  |  |
| Cnih1 | 1.15 | 2.46E-04 | 887.16 | 376.83 | ✓ | NA |  |  |
| Plcl1 | 0.88 | 2.47E-04 | 203.13 | 107.3933 | ✓ | ✓ | ✓ |  |
| Abcf1 | 0.68 | 2.48E-04 | 1274.323 | 786.6967 | ✓ | ✓ |  |  |
| Atp6ap1 | 0.76 | 2.51E-04 | 3972.157 | 2303.657 | ✓ | >100 |  |  |
| Fam195b | 0.96 | 2.53E-04 | 1106.237 | 550.2367 | ✓ | <5 |  |  |
| G3bp2 | 0.72 | 2.56E-04 | 3296.087 | 1973.32 | ✓ | >100 |  |  |
| Cgref1 | 0.91 | 2.66E-04 | 308.3067 | 159.6867 | ✓ | NA |  |  |
| Slc35e1 | 0.63 | 2.74E-04 | 698.37 | 447.2067 | ✓ | <5/>100 |  |  |
| Slc24a2 | 0.61 | 2.74E-04 | 3619.533 | 2354.457 | ✓ | >100 |  |  |
| Supt4a | 1.06 | 2.93E-04 | 559.6867 | 256.5367 | ✓ | NA |  |  |
| Arf1 | 0.87 | 2.99E-04 | 3511.007 | 1869.167 | ✓ | >100 |  |  |
| Chp1 | 0.88 | 3.00E-04 | 1897.157 | 1009.077 | ✓ | NA |  |  |
| Zmat2 | 0.99 | 3.03E-04 | 2444.21 | 1186.443 | ✓ | <5 |  |  |
| Ppargc1a | 0.96 | 3.12E-04 | 883.72 | 438.67 | ✓ | ✓ |  |  |
| **Nts** | **1.47** | **3.40E-04** | **81.83** | **25.08333** | **✓** | **✓** | **✓** | **✓** |
| Tceal8 | 1.05 | 3.41E-04 | 473.6733 | 217.14 | ✓ | ✓ | ✓ |  |
| Fam58b | 1.16 | 3.42E-04 | 164.44 | 68.34 | ✓ | <5 |  |  |
| Cdc5l | 0.78 | 3.53E-04 | 723.5967 | 415.57 | ✓ | ✓ |  |  |
| Esrra | 0.89 | 3.63E-04 | 1153.583 | 607.19 | ✓ | ✓ |  |  |
| Gjc2 | 1.12 | 3.78E-04 | 375.46 | 163.3933 | ✓ | NA |  |  |
| Apoa2 | 1.63 | 3.99E-04 | 25.18 | 6.5 | ✓ | <5 |  |  |
| Atp6v1g1 | 0.9 | 4.02E-04 | 934.7 | 486.4667 | ✓ | <5 |  |  |
| Htr1b | 1.62 | 4.11E-04 | 38.77333 | 10.05667 | ✓ | ✓ |  |  |
| Apbb1 | 0.67 | 4.23E-04 | 3730.757 | 2320.99 | ✓ | >100 |  |  |
| Apol8 | 1.23 | 4.39E-04 | 144.6133 | 56.91667 | ✓ | <5 |  |  |
| G0s2 | 1.25 | 4.67E-04 | 95.10333 | 36.44667 | ✓ | ✓ |  |  |
| Paqr7 | 0.97 | 4.83E-04 | 776.7967 | 381.5867 | ✓ | <5 |  |  |
| Slc25a39 | 0.85 | 4.84E-04 | 921.8667 | 501.1333 | ✓ | ✓ |  |  |
| **Rora** | **1.1** | **4.95E-04** | **1535.063** | **680.19** | **✓** | **✓** | **✓** | **✓** |
| Wnt9b | 1.56 | 4.99E-04 | 28.17 | 7.94 | ✓ | <5 |  |  |
| Fgf12 | 1.16 | 5.29E-04 | 1472.06 | 618.8333 | ✓ | ✓ | ✓ |  |
| Lgi3 | 0.69 | 5.39E-04 | 2366.433 | 1448.927 | ✓ | <5 |  |  |
| Sac3d1 | 1.15 | 5.40E-04 | 171.9333 | 71.91333 | ✓ | NA |  |  |
| Elmo1 | 1.09 | 5.44E-04 | 1636.043 | 725.8767 | ✓ | <5 |  |  |
| Csk | 0.67 | 5.45E-04 | 571.7067 | 357.7767 | ✓ | <5 |  |  |
| Grm1 | 1.38 | 5.49E-04 | 864.7967 | 295.3433 | ✓ | ✓ |  |  |
| Noa1 | 0.66 | 5.55E-04 | 263.7533 | 165.3467 | ✓ | NA |  |  |
| M6pr | 0.63 | 5.59E-04 | 1182.927 | 756.2 | ✓ | ✓ |  |  |
| Praf2 | 1.08 | 5.61E-04 | 923.4167 | 414.9967 | ✓ | >100 |  |  |
| Zbtb7a | 0.91 | 5.61E-04 | 1189.33 | 613.8433 | ✓ | <5/>50 |  |  |
| Tspan12 | 0.84 | 5.63E-04 | 221.7667 | 119.69 | ✓ | <5 |  |  |
| Olfm3 | 1.01 | 5.87E-04 | 455.1633 | 216.8167 | ✓ | NA |  |  |
| Znhit1 | 0.85 | 6.00E-04 | 558.29 | 302.75 | ✓ | ✓ |  |  |
| Slc5a1 | 2.19 | 6.10E-04 | 22.69667 | 1.003333 | ✓ | ✓ | ✓ |  |
| Maf | 0.85 | 6.11E-04 | 514.4 | 278.7067 | ✓ | NA |  |  |
| Abcg1 | 0.74 | 6.12E-04 | 483.5333 | 282.0833 | ✓ | ✓ |  |  |
| Tomm22 | 0.92 | 6.22E-04 | 806.0767 | 412.58 | ✓ | ✓ |  |  |
| Atp2a3 | 1.72 | 6.22E-04 | 493.4833 | 115.8267 | ✓ | ✓ |  |  |
| Lrfn3 | 0.81 | 6.22E-04 | 201.8967 | 111.81 | ✓ | <5 |  |  |
| Mapk6 | 0.79 | 6.55E-04 | 1043.05 | 592.1533 | ✓ | <5/>50 |  |  |
| Unc119 | 0.9 | 7.00E-04 | 559.1533 | 290.1333 | ✓ | <5 |  |  |
| Pdcd2 | 0.79 | 7.01E-04 | 252.2967 | 143.34 | ✓ | ✓ |  |  |
| Arl8b | 0.75 | 7.01E-04 | 1564.757 | 910.5367 | ✓ | ✓ |  |  |
| Tigd2 | 0.92 | 7.27E-04 | 203.0833 | 103.9833 | ✓ | <5 |  |  |
| Ptger4 | 1.56 | 7.49E-04 | 30.26667 | 8.606667 | ✓ | ✓ |  |  |
| Pgr15l | 1.47 | 7.49E-04 | 26.95667 | 8.676667 | ✓ | <5 |  |  |
| Plcl2 | 0.67 | 7.59E-04 | 776.8 | 483.1733 | ✓ | <5 |  |  |
| AsNA1 | 0.9 | 7.66E-04 | 934.27 | 483.12 | ✓ | >100 |  |  |
| Cabp1 | 0.97 | 8.06E-04 | 1818.407 | 896.1433 | ✓ | <5 |  |  |
| Pura | 0.78 | 8.11E-04 | 4653.777 | 2662.423 | ✓ | NA |  |  |
| Fxn | 0.93 | 8.91E-04 | 93.88333 | 47.27667 | ✓ | >100 |  |  |
| Nkx6-2 | 1.28 | 9.17E-04 | 363.7667 | 134.8333 | ✓ | <5 |  |  |
| Opn3 | 1.01 | 9.25E-04 | 84.17667 | 40.46667 | ✓ | ✓ |  |  |
| Isl1 | 1.79 | 9.29E-04 | 55.11 | 11.6 | ✓ | ✓ |  |  |
| Il17d | 0.85 | 9.39E-04 | 119.14 | 63.30333 | ✓ | <5 |  |  |
| Vegfb | 0.6 | 9.45E-04 | 637.6233 | 415.87 | ✓ | <5 |  |  |
| Zbtb7b | 0.54 | 9.74E-04 | 288.44 | 197.47 | ✓ | ✓ |  |  |
| Cox8b | 2.08 | 9.75E-04 | 16.19333 | 1.073333 | ✓ | ✓ |  |  |
| Lrrn1 | 1.01 | 9.89E-04 | 629.9467 | 298.37 | ✓ | ✓ |  |  |
| Zfp536 | 1.01 | 9.96E-04 | 507.0133 | 241.96 | ✓ | <5 |  |  |
| Aqp6 | 2 | 1.01E-03 | 34.78667 | 3.96 | ✓ | NA |  |  |
| Tsc22d3 | 0.79 | 1.02E-03 | 1926.167 | 1089.613 | ✓ | ✓ |  |  |
| Pdlim2 | 1.44 | 1.02E-03 | 284.85 | 88.49333 | ✓ | ✓ | ✓ |  |
| Drap1 | 0.91 | 1.02E-03 | 1440.077 | 743.3333 | ✓ | >100 |  |  |
| Atp6v0e2 | 0.92 | 1.04E-03 | 3619.613 | 1846.297 | ✓ | >100 |  |  |
| Tm9sf3 | 0.6 | 1.07E-03 | 1918.803 | 1256.297 | ✓ | NA |  |  |
| Glra2 | 1.13 | 1.08E-03 | 195.2333 | 82.62333 | ✓ | ✓ | ✓ |  |
| Tnfrsf21 | 0.93 | 1.08E-03 | 1061.733 | 534.5933 | ✓ | >100 |  |  |
| Tmco3 | 0.54 | 1.16E-03 | 480.0133 | 329.0967 | ✓ | ✓ |  |  |
| Rltpr | 0.52 | 1.16E-03 | 906.9067 | 628.8533 | ✓ | ✓ | ✓ |  |
| Homer3 | 1.9 | 1.18E-03 | 1046.507 | 166.94 | ✓ | ✓ |  |  |
| Akr1b3 | 0.72 | 1.18E-03 | 417.51 | 247.77 | ✓ | <5 |  |  |
| Orai1 | 1.07 | 1.18E-03 | 86.63667 | 38.39333 | ✓ | <5 |  |  |
| Zfp771 | 0.71 | 1.23E-03 | 452.27 | 271.2467 | ✓ | ✓ |  |  |
| Kcnd2 | 0.58 | 1.26E-03 | 934.73 | 619.31 | ✓ | ✓ |  |  |
| Shroom2 | 0.76 | 1.27E-03 | 1078.467 | 627.3033 | ✓ | ✓ |  |  |
| Hoxc4 | 1.65 | 1.28E-03 | 17.80667 | 4.353333 | ✓ | <5 |  |  |
| Scp2 | 0.81 | 1.30E-03 | 1188.877 | 657.9433 | ✓ | ✓ |  |  |
| Taf10 | 0.83 | 1.34E-03 | 630.9633 | 345.1 | ✓ | NA |  |  |
| Arl6ip5 | 1.01 | 1.34E-03 | 1394.297 | 656.8667 | ✓ | >100 |  |  |
| Tmem147 | 0.65 | 1.43E-03 | 765.3667 | 483 | ✓ | ✓ |  |  |
| **Nos1** | **0.98** | **1.45E-03** | **955.4967** | **461.21** | **✓** | **✓** | **✓** | **✓** |
| Msl3l2 | 0.91 | 1.46E-03 | 139.2633 | 69.96 | ✓ | <5 |  |  |
| Nfatc2 | 0.86 | 1.47E-03 | 390.91 | 208.89 | ✓ | <5 |  |  |
| Nr2f6 | 0.72 | 1.54E-03 | 547.3767 | 326.95 | ✓ | <5 |  |  |
| Tom1l2 | 0.76 | 1.57E-03 | 4047.91 | 2345.077 | ✓ | >100 |  |  |
| Hcrtr2 | 1.09 | 1.58E-03 | 60.08667 | 26.91667 | ✓ | <5 |  |  |
| Myh7b | 0.95 | 1.61E-03 | 239.2433 | 119.16 | ✓ | NA |  |  |
| Tm9sf2 | 0.63 | 1.61E-03 | 1083.71 | 693.76 | ✓ | >100 |  |  |
| Polr2g | 0.81 | 1.64E-03 | 754.91 | 418.9867 | ✓ | NA |  |  |
| Slc35d3 | 1.15 | 1.65E-03 | 33.30667 | 14.09333 | ✓ | ✓ | ✓ |  |
| **Trh** | **1.5** | **1.67E-03** | **132.47** | **37.27** | **✓** | **✓** | **✓** | **✓** |
| Grin3a | 0.66 | 1.67E-03 | 522.47 | 326.4633 | ✓ | <5 |  |  |
| Pex7 | 0.72 | 1.69E-03 | 320.0933 | 191.8267 | ✓ | <5 |  |  |
| Kat5 | 0.46 | 1.75E-03 | 720.8967 | 519.85 | ✓ | ✓ |  |  |
| Nfkbib | 0.89 | 1.76E-03 | 233.3433 | 120.3733 | ✓ | <5 |  |  |
| Zfpm2 | 0.83 | 1.80E-03 | 219.63 | 120.2067 | ✓ | ✓ |  |  |
| Kcnk15 | 2.01 | 1.85E-03 | 10.45 | 0.593333 | ✓ | <5 |  |  |
| Cdc42 | 0.65 | 1.89E-03 | 3326.693 | 2095.673 | ✓ | >100 |  |  |
| Arf5 | 0.84 | 1.90E-03 | 1332.203 | 721.5167 | ✓ | <5/>100 |  |  |
| Kcnj12 | 0.9 | 1.93E-03 | 131.2967 | 67.07 | ✓ | <5 |  |  |
| Pgrmc1 | 0.87 | 1.99E-03 | 3332.887 | 1759.613 | ✓ | ✓ | ✓ |  |
| Grb2 | 0.85 | 2.02E-03 | 1632.727 | 875.31 | ✓ | ✓ |  |  |
| Gabbr1 | 0.5 | 2.06E-03 | 7030.54 | 4929.707 | ✓ | >100 |  |  |
| GNAi1 | 0.67 | 2.09E-03 | 1456.833 | 900.4267 | ✓ | ✓ | ✓ |  |
| Grm8 | 0.94 | 2.14E-03 | 151.8833 | 75.27 | ✓ | ✓ | ✓ |  |
| Gabarap | 0.93 | 2.14E-03 | 1608.097 | 807.4933 | ✓ | >100 |  |  |
| Grm4 | 0.88 | 2.15E-03 | 588.3333 | 310.1733 | ✓ | <5/>100 |  |  |
| Scg3 | 0.88 | 2.16E-03 | 1990.707 | 1045.157 | ✓ | >100 |  |  |
| Taf13 | 0.91 | 2.17E-03 | 525.6767 | 270.0533 | ✓ | ✓ |  |  |
| Jkamp | 0.77 | 2.17E-03 | 750.8233 | 431.85 | ✓ | ✓ |  |  |
| Nlrp10 | 1.98 | 2.18E-03 | 17.38 | 1.03 | ✓ | <5 |  |  |
| Zmynd19 | 0.82 | 2.19E-03 | 403.9433 | 222.5867 | ✓ | <5 |  |  |
| Doc2b | 0.73 | 2.20E-03 | 556.0867 | 328.03 | ✓ | ✓ |  |  |
| **En2** | **1.14** | **2.21E-03** | **186.8033** | **78.50333** | **✓** | **✓** | **✓** | **✓** |
| Gnb1 | 0.62 | 2.21E-03 | 5601.103 | 3601.1 | ✓ | <5 |  |  |
| Arl8a | 0.66 | 2.24E-03 | 1744.92 | 1085.063 | ✓ | >100 |  |  |
| Mbp | 1.01 | 2.24E-03 | 23680.86 | 11094.05 | ✓ | >100 |  |  |
| Casz1 | 1.34 | 2.25E-03 | 144.3333 | 50.28 | ✓ | ✓ |  |  |
| Mlf2 | 0.64 | 2.41E-03 | 3581.83 | 2267.243 | ✓ | NA |  |  |
| Gabrg1 | 0.81 | 2.56E-03 | 329.9467 | 183.72 | ✓ | ✓ | ✓ |  |
| Htr1d | 1.39 | 2.73E-03 | 27.95667 | 8.99 | ✓ | <5 |  |  |
| Sbds | 0.67 | 2.78E-03 | 855.57 | 526.6 | ✓ | >100 |  |  |
| Nfic | 0.76 | 2.79E-03 | 1088.21 | 629.36 | ✓ | ✓ |  |  |
| Nectin4 | 1.11 | 2.83E-03 | 151.41 | 64.64 | ✓ | NA |  |  |
| Zfp423 | 0.72 | 2.87E-03 | 401.5667 | 238.1167 | ✓ | <5 |  |  |
| Cox16 | 1.21 | 2.90E-03 | 107.1167 | 41.12667 | ✓ | NA |  |  |
| Tac2 | 1.28 | 2.95E-03 | 59.62333 | 20.99333 | ✓ | ✓ |  |  |
| Slc17a8 | 1.06 | 3.08E-03 | 86.48667 | 38.95333 | ✓ | ✓ |  |  |
| Slc7a14 | 0.44 | 3.09E-03 | 951.87 | 700.5567 | ✓ | ✓ |  |  |
| Tom1 | 0.5 | 3.11E-03 | 560.3067 | 395.5133 | ✓ | NA |  |  |
| Cops5 | 0.5 | 3.13E-03 | 946.6433 | 669.03 | ✓ | >100 |  |  |
| Slc12a8 | 1.46 | 3.17E-03 | 33.21 | 9.506667 | ✓ | <5 |  |  |
| Ifit2 | 0.97 | 3.23E-03 | 146.37 | 69.35333 | ✓ | <5 |  |  |
| Nppc | 1.3 | 3.31E-03 | 58.18333 | 19.61333 | ✓ | <5 |  |  |
| Tacr1 | 0.92 | 3.34E-03 | 116.58 | 59.58 | ✓ | <5 |  |  |
| Sema7a | 0.88 | 3.41E-03 | 2001.83 | 1044.547 | ✓ | ✓ |  |  |
| Faf2 | 0.52 | 3.48E-03 | 628.5833 | 432.62 | ✓ | <5 |  |  |
| Irx5 | 1.18 | 3.50E-03 | 39.05 | 15.36333 | ✓ | <5 |  |  |
| Rxra | 0.62 | 3.54E-03 | 528.46 | 338.7233 | ✓ | ✓ |  |  |
| App | 0.93 | 3.55E-03 | 16463.24 | 8269.9 | ✓ | >100 |  |  |
| Dirc2 | 0.7 | 3.56E-03 | 567.68 | 341.44 | ✓ | ✓ |  |  |
| Fblim1 | 1.3 | 3.65E-03 | 46.82667 | 16.43333 | ✓ | <5 |  |  |
| Txndc17 | 0.82 | 3.67E-03 | 351.3 | 192.5233 | ✓ | ✓ |  |  |
| Reep2 | 0.42 | 3.67E-03 | 1510.333 | 1124.58 | ✓ | NA |  |  |
| P2ry1 | 1.02 | 3.71E-03 | 117.3633 | 53.76 | ✓ | ✓ |  |  |
| Npy | 1.02 | 3.78E-03 | 225.1633 | 103.5067 | ✓ | ✓ |  |  |
| Arhgap12 | 0.73 | 3.78E-03 | 889.65 | 526.33 | ✓ | ✓ | ✓ |  |
| Npffr1 | 1.79 | 3.80E-03 | 13.24667 | 1.736667 | ✓ | NA |  |  |
| 1190002N15Rik | 0.84 | 3.80E-03 | 378.8233 | 204.0167 | ✓ | <5 |  |  |
| Atg3 | 0.7 | 3.81E-03 | 517.9067 | 310.7933 | ✓ | ✓ | ✓ |  |
| Armc1 | 0.63 | 3.85E-03 | 989.36 | 631.12 | ✓ | NA |  |  |
| Zbtb11 | 0.53 | 3.87E-03 | 2622.32 | 1801.423 | ✓ | <5 |  |  |
| Derl1 | 0.62 | 3.91E-03 | 876.5 | 561.78 | ✓ | <5 |  |  |
| Sarnp | 0.74 | 3.94E-03 | 514.4467 | 300.37 | ✓ | <5 |  |  |
| Mrgpre | 1.07 | 3.98E-03 | 162.0667 | 71.53667 | ✓ | <5 |  |  |
| Ehd1 | 0.62 | 4.06E-03 | 468.8833 | 300.87 | ✓ | ✓ |  |  |
| Nup50 | 0.64 | 4.07E-03 | 719.2733 | 455.9467 | ✓ | ✓ |  |  |
| Wnt7a | 1 | 4.08E-03 | 152.1333 | 70.67333 | ✓ | <5 |  |  |
| Gas6 | 0.46 | 4.08E-03 | 1797.367 | 1299.04 | ✓ | >100 |  |  |
| Atp6v1g2 | 0.89 | 4.12E-03 | 4794.81 | 2476.973 | ✓ | NA |  |  |
| Cox8a | 0.99 | 4.18E-03 | 2475.88 | 1177.637 | ✓ | <5 |  |  |
| Kcnn1 | 0.78 | 4.47E-03 | 220.86 | 124.9933 | ✓ | <5 |  |  |
| KpNA2 | 0.59 | 4.56E-03 | 311.2033 | 204.78 | ✓ | <5 |  |  |
| Ascl4 | 1.84 | 4.58E-03 | 38.84667 | 3.14 | ✓ | NA |  |  |
| Cux1 | 0.66 | 4.61E-03 | 474.2567 | 295.85 | ✓ | ✓ |  |  |
| Rgmb | 0.78 | 4.62E-03 | 1384.78 | 783.9833 | ✓ | <5 |  |  |
| Gsk3b | 0.67 | 5.01E-03 | 2130.727 | 1316.22 | ✓ | >100 |  |  |
| Dcbld1 | 0.72 | 5.13E-03 | 134.3233 | 79.09667 | ✓ | <5 |  |  |
| Slc22a23 | 0.45 | 5.24E-03 | 745.4667 | 540.0867 | ✓ | ✓ |  |  |
| Bmp2 | 0.99 | 5.46E-03 | 66.47 | 31.37333 | ✓ | <5 |  |  |
| Dap | 1.25 | 5.62E-03 | 119.93 | 42.99333 | ✓ | <5 |  |  |
| Wnt3 | 0.95 | 5.64E-03 | 115.7867 | 57.22667 | ✓ | <5 |  |  |
| Foxj1 | 0.84 | 5.67E-03 | 141.7533 | 76.33 | ✓ | ✓ |  |  |
| GriNA | 0.54 | 5.82E-03 | 4422.32 | 3007.477 | ✓ | ✓ |  |  |
| Kcnip4 | 0.8 | 5.88E-03 | 826.3267 | 459.7567 | ✓ | ✓ | ✓ |  |
| Pole4 | 0.75 | 5.92E-03 | 358.2867 | 206.09 | ✓ | ✓ |  |  |
| Emb | 0.67 | 6.10E-03 | 383.2767 | 236.9467 | ✓ | ✓ |  |  |
| Elmod1 | 0.48 | 6.49E-03 | 1536.313 | 1095.003 | ✓ | >100 |  |  |
| Lrrn3 | 0.78 | 6.51E-03 | 550.69 | 311.5733 | ✓ | <5/>100 |  |  |
| Fgf11 | 0.75 | 6.51E-03 | 838.5267 | 485.3233 | ✓ | NA |  |  |
| Ctnnb1 | 0.53 | 6.61E-03 | 4017.4 | 2754.667 | ✓ | <5/>100 |  |  |
| MNAt1 | 0.62 | 6.87E-03 | 202.0133 | 129.69 | ✓ | <5 |  |  |
| Ppp1r1b | 0.95 | 7.08E-03 | 769.6367 | 376.86 | ✓ | ✓ |  |  |
| Dpf3 | 0.96 | 7.25E-03 | 66.08 | 31.29 | ✓ | ✓ |  |  |
| Tspan7 | 0.86 | 7.36E-03 | 6819.003 | 3611.833 | ✓ | >100 |  |  |
| Negr1 | 0.73 | 7.50E-03 | 1527.843 | 896.31 | ✓ | <5 |  |  |
| Spry4 | 0.72 | 7.65E-03 | 226.7533 | 134.1567 | ✓ | <5 |  |  |
| Gtf2b | 0.7 | 7.80E-03 | 425.21 | 254.9833 | ✓ | ✓ |  |  |
| Nrg1 | 0.77 | 8.09E-03 | 74.47 | 42.90333 | ✓ | ✓ |  |  |
| Tmeff2 | 0.6 | 8.11E-03 | 788.2367 | 513.3767 | ✓ | >100 |  |  |
| Sema3e | 0.68 | 8.34E-03 | 257.4333 | 158.27 | ✓ | ✓ |  |  |
| Btbd2 | 0.46 | 8.50E-03 | 711.68 | 513.9267 | ✓ | ✓ |  |  |
| **S100b** | **1.45** | **8.54E-03** | **2834.257** | **747.01** | **✓** | **✓** | **✓** | **✓** |
| Dscr3 | 0.51 | 8.63E-03 | 583.93 | 408.8033 | ✓ | NA |  |  |
| Btbd3 | 0.46 | 8.66E-03 | 1330.523 | 962.0767 | ✓ | ✓ |  |  |
| Aatf | 0.68 | 8.66E-03 | 211.9067 | 130.2833 | ✓ | <5 |  |  |
| CntNAp4 | 0.74 | 8.79E-03 | 528.0233 | 310.1667 | ✓ | <5 |  |  |
| Oxa1l | 0.39 | 8.89E-03 | 509.2467 | 386.9 | ✓ | NA |  |  |
| Arhgef4 | 0.44 | 9.09E-03 | 2563.777 | 1880.757 | ✓ | NA |  |  |
| Unc5b | 0.69 | 9.17E-03 | 448.47 | 271.7367 | ✓ | ✓ | ✓ |  |
| Cacng4 | 0.87 | 9.22E-03 | 718.96 | 376.19 | ✓ | ✓ |  |  |
| Nkx2-2 | 0.83 | 9.25E-03 | 89.52333 | 48.72333 | ✓ | ✓ |  |  |
| Atg5 | 0.65 | 9.31E-03 | 301.96 | 188.3633 | ✓ | ✓ |  |  |
| Lgr5 | 0.86 | 9.42E-03 | 105.37 | 56.86667 | ✓ | ✓ |  |  |
| Slc8a3 | 0.62 | 9.43E-03 | 371.51 | 237.8933 | ✓ | >100 |  |  |
| Ptprm | 0.84 | 9.43E-03 | 519.17 | 278.2567 | ✓ | <5 |  |  |
| Gtf2e2 | 0.73 | 9.43E-03 | 237.2 | 138.6933 | ✓ | NA |  |  |
| Cort | 1.17 | 9.58E-03 | 40.36667 | 15.66 | ✓ | <5 |  |  |
| Stx18 | 1.23 | 9.86E-03 | 702.75 | 256.72 | ✓ | <5 |  |  |
| Dtx1 | 0.52 | 9.87E-03 | 822.4733 | 565.8933 | ✓ | <5 |  |  |
| Adcyap1 | 0.91 | 9.98E-03 | 279.0333 | 139.7133 | ✓ | <5/>100 |  |  |
| Kcnk12 | 0.97 | 0.0101 | 95.35 | 44.81333 | ✓ | NA |  |  |
| Lsamp | 0.71 | 1.01E-02 | 3144.223 | 1881.777 | ✓ | ✓ | ✓ |  |
| Bnip3l | 0.74 | 1.02E-02 | 1437.62 | 835.99 | ✓ | ✓ | ✓ |  |
| Psip1 | 0.42 | 1.02E-02 | 3052.007 | 2278.067 | ✓ | <5 |  |  |
| Tfap2b | 1.22 | 1.03E-02 | 193.18 | 70.76333 | ✓ | NA |  |  |
| Crtam | 1.61 | 1.05E-02 | 47.90667 | 7.903333 | ✓ | <5 |  |  |
| Cbx4 | 0.69 | 1.05E-02 | 680.4433 | 411.8333 | ✓ | <5 |  |  |
| Ngf | 1.26 | 1.06E-02 | 28.19667 | 9.54 | ✓ | <5 |  |  |
| Cntn1 | 0.63 | 1.07E-02 | 3445.753 | 2183.01 | ✓ | ✓ | ✓ |  |
| Zfp787 | 0.65 | 1.07E-02 | 200.1033 | 124.0633 | ✓ | <5 |  |  |
| Tbl3 | 0.54 | 1.13E-02 | 433.35 | 296.0567 | ✓ | ✓ |  |  |
| Gjb1 | 1.1 | 1.14E-02 | 200.7367 | 83.43 | ✓ | <5 |  |  |
| Zfp597 | 0.57 | 1.15E-02 | 285.8433 | 189.5833 | ✓ | ✓ |  |  |
| Olig1 | 1.04 | 1.18E-02 | 1514.32 | 672.6267 | ✓ | <5 |  |  |
| Arhgdia | 0.66 | 1.18E-02 | 2164.75 | 1336.117 | ✓ | >100 |  |  |
| Irx4 | 1.61 | 1.19E-02 | 13.65 | 1.836667 | ✓ | <5 |  |  |
| Gata2 | 0.73 | 1.20E-02 | 155.69 | 90.44 | ✓ | >100 |  |  |
| Abcf3 | 0.59 | 1.21E-02 | 1291.573 | 849.0333 | ✓ | <5 |  |  |
| Lpar1 | 1.04 | 1.22E-02 | 351.8667 | 154.2467 | ✓ | ✓ | ✓ |  |
| Gdf10 | 0.95 | 1.22E-02 | 65.18333 | 30.48333 | ✓ | <5 |  |  |
| Tsc22d1 | 0.58 | 1.23E-02 | 4098.153 | 2704.167 | ✓ | >100 |  |  |
| Leng1 | 0.62 | 1.24E-02 | 193.67 | 123.6933 | ✓ | <5 |  |  |
| Nrip3 | 0.68 | 1.25E-02 | 2677.5 | 1638.907 | ✓ | <5 |  |  |
| Fam43b | 0.74 | 1.27E-02 | 285.14 | 166.6567 | ✓ | NA |  |  |
| Atf4 | 0.49 | 1.27E-02 | 1722.967 | 1214.483 | ✓ | >100 |  |  |
| Socs5 | 0.54 | 1.28E-02 | 1493.47 | 1015.81 | ✓ | >100 |  |  |
| Hoxb4 | 1.52 | 1.31E-02 | 10.94 | 1.91 | ✓ | ✓ |  |  |
| Rcan1 | 0.57 | 1.31E-02 | 616.48 | 407.9233 | ✓ | <5 |  |  |
| Asic1 | 0.45 | 1.32E-02 | 761.1767 | 556.6133 | ✓ | NA |  |  |
| Bud31 | 0.56 | 1.34E-02 | 435.7 | 291.9833 | ✓ | <5 |  |  |
| Lrrc8d | 0.65 | 1.36E-02 | 315.34 | 195.3333 | ✓ | NA |  |  |
| Dgkg | 0.72 | 1.37E-02 | 520.5233 | 307.0433 | ✓ | ✓ |  |  |
| Gtf2f2 | 0.79 | 1.37E-02 | 133.6567 | 72.79333 | ✓ | <5 |  |  |
| Pth1r | 0.73 | 1.38E-02 | 133.9067 | 77.89 | ✓ | ✓ |  |  |
| Gnl3l | 0.52 | 1.38E-02 | 2335.713 | 1614.063 | ✓ | >100 |  |  |
| Kpnb1 | 0.41 | 1.39E-02 | 1753.96 | 1317.593 | ✓ | ✓ | ✓ |  |
| Kcnj5 | 0.97 | 1.40E-02 | 37.44 | 17.95333 | ✓ | <5 |  |  |
| NAcc1 | 0.44 | 1.46E-02 | 1029.327 | 750.9267 | ✓ | ✓ |  |  |
| Abcf2 | 0.6 | 1.46E-02 | 995.8333 | 646.71 | ✓ | >100 |  |  |
| Atp5g2 | 0.85 | 1.50E-02 | 732.2367 | 386.09 | ✓ | NA |  |  |
| Lrrtm3 | 0.72 | 1.50E-02 | 362.2667 | 214.0167 | ✓ | <5 |  |  |
| Gid8 | 0.68 | 1.51E-02 | 466.18 | 282.4667 | ✓ | NA |  |  |
| Sdc3 | 0.61 | 1.56E-02 | 2516.153 | 1618.02 | ✓ | >100 |  |  |
| Lrp12 | 0.61 | 1.56E-02 | 394.9633 | 254.4167 | ✓ | <5 |  |  |
| Abtb2 | 0.62 | 1.56E-02 | 178.7867 | 112.9767 | ✓ | <5 |  |  |
| Serinc1 | 0.51 | 1.56E-02 | 4858.543 | 3382.35 | ✓ | >100 |  |  |
| Taf9 | 0.55 | 1.60E-02 | 425.1967 | 285.3433 | ✓ | <5 |  |  |
| Thra | 0.54 | 1.64E-02 | 6484.623 | 4401.097 | ✓ | ✓ |  |  |
| 4930452B06Rik | 0.73 | 1.65E-02 | 139.66 | 81.88 | ✓ | ✓ |  |  |
| Tpr | 0.76 | 1.66E-02 | 1582.54 | 900.8633 | ✓ | ✓ |  |  |
| Btg1 | 0.64 | 1.66E-02 | 380.7967 | 238.3 | ✓ | ✓ |  |  |
| Lrsam1 | 0.64 | 1.68E-02 | 613.7933 | 385.69 | ✓ | ✓ | ✓ |  |
| Mapk8ip1 | 0.42 | 1.71E-02 | 4261.11 | 3176.757 | ✓ | >100 |  |  |
| Srpr | 0.49 | 1.73E-02 | 706.9833 | 500.5733 | ✓ | ✓ | ✓ |  |
| Tspan2 | 0.73 | 1.74E-02 | 1686.29 | 984.9367 | ✓ | <5/>100 |  |  |
| Zhx1 | 0.53 | 1.75E-02 | 641.5367 | 437.2367 | ✓ | ✓ |  |  |
| Cbx5 | 0.46 | 1.77E-02 | 2102.827 | 1521.293 | ✓ | ✓ |  |  |
| Yaf2 | 0.63 | 1.77E-02 | 751.4367 | 474.8033 | ✓ | ✓ |  |  |
| Cd81 | 0.73 | 1.77E-02 | 2664.823 | 1555.86 | ✓ | >100 |  |  |
| Kdelr2 | 0.57 | 1.79E-02 | 282.05 | 185.7267 | ✓ | <5 |  |  |
| Cadm3 | 0.6 | 1.80E-02 | 3352.033 | 2181.43 | ✓ | >100 |  |  |
| Zhx3 | 0.43 | 1.80E-02 | 668.62 | 491.46 | ✓ | <5 |  |  |
| Mpnd | 0.55 | 1.80E-02 | 1304.997 | 877.38 | ✓ | <5 |  |  |
| Wif1 | 1.18 | 1.81E-02 | 17.05667 | 6.406667 | ✓ | <5 |  |  |
| Ehd4 | 0.61 | 1.82E-02 | 220.6967 | 142.7267 | ✓ | ✓ |  |  |
| Agrp | 1.52 | 1.82E-02 | 16.79333 | 3.023333 | ✓ | <5 |  |  |
| B3galt4 | 0.75 | 1.84E-02 | 48.69 | 27.87667 | ✓ | <5 |  |  |
| Foxb1 | 1.25 | 1.86E-02 | 48.4 | 15.87 | ✓ | ✓ |  |  |
| Nxph2 | 1.11 | 1.89E-02 | 34.47667 | 14.32333 | ✓ | <5 |  |  |
| Igfbp4 | 0.68 | 1.90E-02 | 588.56 | 355.8733 | ✓ | ✓ | ✓ |  |
| Slc12a5 | 0.53 | 1.90E-02 | 6176.3 | 4233.6 | ✓ | >100 |  |  |
| Tspan4 | 0.75 | 1.94E-02 | 258.66 | 147.3567 | ✓ | <5 |  |  |
| Zfp37 | 0.58 | 1.95E-02 | 188.51 | 123.8067 | ✓ | <5 |  |  |
| Bok | 0.79 | 1.97E-02 | 318.3867 | 176.8767 | ✓ | ✓ |  |  |
| Polr3h | 0.66 | 2.00E-02 | 469.4167 | 289.8133 | ✓ | ✓ |  |  |
| Atp2a1 | 1.24 | 2.04E-02 | 22.76 | 7.46 | ✓ | NA |  |  |
| Elfn1 | 0.68 | 2.06E-02 | 227.48 | 137.4667 | ✓ | ✓ | ✓ |  |
| Prmt5 | 0.34 | 2.07E-02 | 564.1633 | 443.64 | ✓ | <5 |  |  |
| Clcn5 | 0.58 | 2.08E-02 | 360.38 | 239.8167 | ✓ | ✓ | ✓ |  |
| Fam19a2 | 0.81 | 2.09E-02 | 589.7867 | 321.5733 | ✓ | <5 |  |  |
| Bcl2 | 0.68 | 2.12E-02 | 263.03 | 158.7167 | ✓ | ✓ |  |  |
| Uso1 | 0.44 | 2.12E-02 | 1112.87 | 813.2633 | ✓ | NA |  |  |
| Sorl1 | 0.69 | 2.13E-02 | 3487.53 | 2106.27 | ✓ | >100 |  |  |
| Arhgap26 | 0.65 | 2.19E-02 | 1179.553 | 732.74 | ✓ | ✓ |  |  |
| Slc25a2 | 1.51 | 2.20E-02 | 9.623333 | 1.536667 | ✓ | <5 |  |  |
| Ogfrl1 | 0.71 | 2.21E-02 | 2044.653 | 1213.25 | ✓ | ✓ |  |  |
| Cyb5d2 | 0.62 | 2.21E-02 | 137.5833 | 87.32667 | ✓ | <5 |  |  |
| Nhlh2 | 0.75 | 2.29E-02 | 91.50333 | 53.29333 | ✓ | ✓ |  |  |
| Xpr1 | 0.47 | 2.29E-02 | 886.0133 | 632.0067 | ✓ | ✓ |  |  |
| Hoxb2 | 1.46 | 2.35E-02 | 9.966667 | 1.72 | ✓ | ✓ |  |  |
| Nectin1 | 0.54 | 2.37E-02 | 557.7067 | 378.82 | ✓ | NA |  |  |
| Smyd3 | 0.53 | 2.37E-02 | 403.4133 | 275.2067 | ✓ | <5 |  |  |
| Chrac1 | 0.59 | 2.51E-02 | 141.29 | 91.48 | ✓ | ✓ |  |  |
| C3 | 1.17 | 2.52E-02 | 21.53333 | 8.116667 | ✓ | <5 |  |  |
| Creld1 | 0.55 | 2.58E-02 | 1141.957 | 769.56 | ✓ | >100 |  |  |
| Irs1 | 0.61 | 2.59E-02 | 307.2367 | 197.0033 | ✓ | <5 |  |  |
| Stat5b | 0.54 | 2.64E-02 | 466.7167 | 316.8133 | ✓ | ✓ |  |  |
| Hspa12a | 0.62 | 2.68E-02 | 2730.873 | 1745.787 | ✓ | <5 |  |  |
| Acsf3 | 0.53 | 2.75E-02 | 265.5467 | 181.6433 | ✓ | ✓ |  |  |
| Gucy1a3 | 0.59 | 2.75E-02 | 671.4467 | 439.7667 | ✓ | ✓ | ✓ |  |
| Rbm43 | 0.49 | 2.81E-02 | 242.7833 | 171.47 | ✓ | ✓ |  |  |
| Mafa | 1.44 | 2.81E-02 | 13.48 | 2.066667 | ✓ | NA |  |  |
| G3bp1 | 0.35 | 2.82E-02 | 886.06 | 693.49 | ✓ | ✓ |  |  |
| Hoxb3 | 1.41 | 2.99E-02 | 12.2 | 2.283333 | ✓ | <5 |  |  |
| Bloc1s1 | 0.88 | 3.00E-02 | 172.83 | 86.1 | ✓ | <5 |  |  |
| Tceal5 | 0.57 | 3.01E-02 | 664.11 | 439.9167 | ✓ | <5 |  |  |
| Svop | 0.48 | 3.04E-02 | 1515.28 | 1072.097 | ✓ | >100 |  |  |
| Esr1 | 0.91 | 3.08E-02 | 56.19 | 26.95 | ✓ | <5 |  |  |
| Camta1 | 0.4 | 3.09E-02 | 4724.483 | 3568.28 | ✓ | <5/>100 |  |  |
| Tmeff1 | 0.58 | 3.10E-02 | 843.09 | 552.85 | ✓ | ✓ | ✓ |  |
| Apbb2 | 0.5 | 3.13E-02 | 538.8633 | 376.8233 | ✓ | ✓ |  |  |
| Jup | 0.65 | 3.13E-02 | 401.95 | 248.3633 | ✓ | ✓ |  |  |
| Calcr | 1.25 | 3.23E-02 | 22.9 | 7.043333 | ✓ | <5 |  |  |
| KcNA5 | 0.94 | 3.27E-02 | 40.52333 | 19.13333 | ✓ | <5 |  |  |
| Sp8 | 1.3 | 3.29E-02 | 26.94333 | 7.903333 | ✓ | <5 |  |  |
| Slc45a1 | 0.4 | 3.30E-02 | 761.2267 | 573.4 | ✓ | ✓ |  |  |
| Jun | 0.59 | 3.30E-02 | 667.1933 | 432.3233 | ✓ | >100 |  |  |
| Scfd2 | 0.56 | 3.32E-02 | 153.8333 | 102.5267 | ✓ | <5 |  |  |
| Stk24 | 0.71 | 3.37E-02 | 460.7367 | 270.9967 | ✓ | ✓ |  |  |
| Slc17a6 | 0.74 | 3.37E-02 | 1073.203 | 615.3 | ✓ | <5>100 |  |  |
| Slc38a10 | 0.38 | 3.41E-02 | 831.0867 | 636.4233 | ✓ | <5/>100 |  |  |
| Homez | 0.55 | 3.41E-02 | 171.94 | 115.0367 | ✓ | >100 |  |  |
| Zmynd12 | 1.41 | 3.42E-02 | 20.06667 | 2.67 | ✓ | NA |  |  |
| Vdr | 1.37 | 3.44E-02 | 11.19333 | 2.06 | ✓ | ✓ |  |  |
| Six3 | 1.4 | 3.44E-02 | 5.78 | 0.706667 | ✓ | >100 |  |  |
| Slc25a38 | 0.47 | 3.48E-02 | 339.3833 | 242.7633 | ✓ | NA |  |  |
| Zfp385b | 0.68 | 3.51E-02 | 227.3233 | 137.1067 | ✓ | ✓ |  |  |
| Atp2a2 | 0.5 | 3.51E-02 | 6564.203 | 4576.683 | ✓ | >100 |  |  |
| Grid1 | 0.61 | 3.59E-02 | 509.1833 | 324.9133 | ✓ | <5 |  |  |
| Sparcl1 | 0.71 | 3.63E-02 | 11640.02 | 6837.187 | ✓ | >100 |  |  |
| Tceal7 | 1.3 | 3.69E-02 | 40.93333 | 10.79333 | ✓ | <5 |  |  |
| Oxtr | 0.69 | 3.70E-02 | 82.26667 | 48.74 | ✓ | ✓ |  |  |
| Sstr5 | 1.22 | 3.81E-02 | 11.68 | 3.533333 | ✓ | <5 |  |  |
| Tcea1 | 0.44 | 3.91E-02 | 938.42 | 689.0533 | ✓ | <5 |  |  |
| Pgf | 0.86 | 3.92E-02 | 68.12667 | 34.02 | ✓ | ✓ |  |  |
| Ifit3b | 0.85 | 3.95E-02 | 51.41667 | 25.64 | ✓ | NA |  |  |
| Slc22a17 | 0.42 | 3.98E-02 | 5498.957 | 4093.96 | ✓ | >100 |  |  |
| Gabre | 0.96 | 4.00E-02 | 24.98 | 12.22333 | ✓ | ✓ | ✓ |  |
| Gpr37 | 0.8 | 4.04E-02 | 405.57 | 218.9667 | ✓ | ✓ |  |  |
| Tmem241 | 0.72 | 4.07E-02 | 71.84333 | 41.20667 | ✓ | <5 |  |  |
| **Nrp2** | **0.42** | **4.10E-02** | **356.91** | **264.8933** | **✓** | **✓** | **✓** | **✓** |
| Slc5a3 | 0.5 | 4.11E-02 | 478.4367 | 334.8233 | ✓ | ✓ |  |  |
| Atf2 | 0.49 | 4.15E-02 | 1090.033 | 764.8333 | ✓ | >100 |  |  |
| Btbd1 | 0.42 | 4.17E-02 | 1382.643 | 1022.57 | ✓ | <5 |  |  |
| Tnfrsf11b | 0.91 | 4.20E-02 | 30.86333 | 14.34667 | ✓ | ✓ |  |  |
| Foxp4 | 0.53 | 4.22E-02 | 493.02 | 334.8267 | ✓ | ✓ | ✓ |  |
| Foxp2 | 0.82 | 4.31E-02 | 429.23 | 229.2567 | ✓ | ✓ | ✓ |  |
| Foxo3 | 0.45 | 4.36E-02 | 695.6433 | 501.12 | ✓ | <5 |  |  |
| Hoxa2 | 1.35 | 4.36E-02 | 6.626667 | 1.046667 | ✓ | <5 |  |  |
| Kcnk13 | 0.79 | 4.39E-02 | 86.76667 | 46.82 | ✓ | ✓ |  |  |
| Pax3 | 1.09 | 4.43E-02 | 22.39333 | 8.916667 | ✓ | ✓ |  |  |
| Cit | 0.5 | 4.43E-02 | 2091.9 | 1460.26 | ✓ | <5/>100 |  |  |
| Nkiras2 | 0.61 | 4.44E-02 | 126.7733 | 80.38 | ✓ | ✓ |  |  |
| Ell3 | 1.26 | 4.47E-02 | 5.14 | 0 | ✓ | <5 |  |  |
| Tada2b | 0.55 | 4.51E-02 | 333.5167 | 222.5767 | ✓ | ✓ |  |  |
| Ucn | 1.3 | 4.54E-02 | 45.24 | 9.57 | ✓ | ✓ |  |  |
| Asgr1 | 1.13 | 4.59E-02 | 57.04333 | 20.16 | ✓ | ✓ |  |  |
| Scarb2 | 0.49 | 4.59E-02 | 350.5933 | 245.33 | ✓ | >100 |  |  |
| Zmat4 | 0.62 | 4.68E-02 | 602.7167 | 381.7967 | ✓ | ✓ |  |  |
| Slc43a2 | 0.49 | 4.68E-02 | 885.3633 | 622.33 | ✓ | ✓ |  |  |
| Bcam | 0.58 | 4.71E-02 | 280.7233 | 183.31 | ✓ | ✓ | ✓ |  |
| Med27 | 0.41 | 4.76E-02 | 172.2067 | 128.5267 | ✓ | NA |  |  |
| Gng11 | 0.89 | 4.78E-02 | 179.9233 | 87.80667 | ✓ | <5/>100 |  |  |
| Kdelr1 | 0.56 | 4.79E-02 | 427.0667 | 282.36 | ✓ | ✓ | ✓ |  |
| Psen2 | 0.41 | 4.88E-02 | 229.2667 | 172.1067 | ✓ | ✓ |  |  |
| Mapk10 | 0.58 | 4.96E-02 | 3243.447 | 2129.17 | ✓ | <5/>100 |  |  |
